# Transcriptomic profiling identifies immunotherapy-responsive phenotypes in microsatellite-stable metastatic colorectal cancer

**DOI:** 10.1038/s41388-026-03861-2

**Published:** 2026-06-16

**Authors:** Tomas Konecny, Nate Zadirako, Arpine Grigoryan, Melina Tamazyan, Sveta Mnatsakanyan, Luiza Stepanyan, Henry Loeffler-Wirth, Sean Bourdelais, Gabriel Mednick, Chloe Delepine, Dhan Chand, Hans Binder

**Affiliations:** 1 Armenian Bioinformatics Institute (ABI), Yerevan, Armenia; 2 Interdisciplinary Centre for Bioinformatics (IZBI), Universität Leipzig, Leipzig, Germany; 3 Institute of Molecular Biology of the National Academy of Sciences of the Republic of Armenia (IMB), Yerevan, Armenia; 4 Agenus Inc., Lexington, MA USA

**Keywords:** Cancer microenvironment, Metastasis

## Abstract

Conventional immune checkpoint inhibitors (ICIs) remain largely ineffective in microsatellite-stable metastatic colorectal cancer (MSS mCRC), where low tumor immunogenicity and molecular heterogeneity across metastatic sites underpin therapeutic resistance. We present a comprehensive transcriptomics analysis of metastatic and primary tumor biopsies from MSS mCRC patients treated with botensilimab (BOT; Fc-enhanced anti–*CTLA-4*) ± balstilimab (BAL; anti–*PD-1)*. Self-organizing map (SOM) machine learning stratified tumors into four molecular types, including a liver-like (LIV) subtype characterized by metabolic reprogramming and immunosuppressive signatures, and proliferative (PRO), inflammatory (INF), and mesenchymal (MES) types concordant with pan-cancer classifications. PRO, INF, and MES types were enriched for epithelial tumor cells, immune cells, and fibroblasts, respectively, defining immune-depleted, immune-enriched, and fibrotic states along a plasticity gradient. We observed treatment-related transcriptomic shifts toward immune-enriched states via upregulation of antigen presentation, T cell recruitment, and cytotoxicity pathways. INF and MES tumor types exhibited improved clinical responses and survival vs PRO and LIV types. This study identified distinct tumor microenvironment states that align along an immunophenotype axis marked by CD74, interferon-γ, and *APOBEC3* expression identified previously for primary CRC. Our findings provide novel insights into molecular correlates of immunotherapy response in MSS mCRC, potentially informing future therapeutic strategies to expand ICI efficacy to historically unresponsive tumors.

## Introduction

Colorectal cancer (CRC) is the second leading cause of cancer-related deaths globally [1]. Despite advances in systemic therapy, patients with metastatic disease, which most commonly involves the liver, lungs, and peritoneum, have a 5-year survival rate of only 13% [1–4]. Although immunotherapy has transformed outcomes for many cancers, its efficacy in CRC remains largely limited to the small subset of tumors with mismatch repair deficiency (dMMR) or microsatellite instability-high (MSI-H) status. These hypermutated tumors harbor high neoantigen loads and exhibit robust immune activation following checkpoint blockade therapy [5, 6]. In contrast, 95% of metastatic CRCs are microsatellite-stable (MSS) and frequently remain refractory to immune checkpoint inhibitors (ICIs).

Several features are associated with this resistance, including low neoantigen load, impaired antigen presentation, an immunosuppressive tumor microenvironment (TME) dominated by regulatory T cells and myeloid suppressor populations, and high molecular heterogeneity across metastatic sites. Consequently, patients with MSS disease derive minimal benefit from conventional checkpoint inhibitors targeting *PD-1* or *CTLA-4*, underscoring the need for new strategies capable of reprogramming these immunologically “cold” tumors [7, 8].

Recent clinical advances, including botensilimab (BOT), a multifunctional Fc-enhanced anti–*CTLA-4* antibody designed to harness novel Fcγ-receptor-dependent mechanisms to enhance T cell priming, activate myeloid cells, and deplete intratumoral regulatory T cells, have shown encouraging clinical activity in immunologically “cold” and treatment-refractory cancers [9–11]. In treatment-refractory MSS mCRC, BOT combined with balstilimab (BAL), an anti–*PD-1* antibody, demonstrated an objective response rate of 22% in heavily pretreated MSS metastatic CRC (mCRC) patients, with a disease control rate of 61% [9]. Notably, clinical benefit was durable and most pronounced in patients without active liver metastases (LM). In the neoadjuvant setting, BOT + BAL produced exceptional pathological responses in locally advanced MSS mCRC, with a novel “inside-out” (serosa-to-mucosa) regression pattern not previously observed with conventional therapies [12]. Although this novel combination strategy has shown promise in extending benefit to patients with MSS mCRC, treatment failure persists in a considerable fraction of patients, and the molecular determinants of response and resistance remain poorly understood.

Here, we present the first comprehensive transcriptomic analysis of metastatic and primary tumor biopsies from patients with MSS mCRC treated with BOT ± BAL in a phase I clinical trial. Using self-organizing map (SOM) machine learning and ecotyping, we define the distinct molecular states of the TME that underlie immune responsiveness and provide mechanistic insights by which BOT remodels the TME to achieve durable clinical benefit.

## Materials and methods

### Patient data and bulk mRNA sequencing

Tumor samples were collected from patients with advanced refractory MSS mCRC treated with BOT ± BAL as part of a clinical trial (NCT03860272), as described previously [9–11] (see also Supplementary Methods). All patient biospecimens were collected and used in compliance with the Agenus Inc. C-800-01 study protocol, the Declaration of Helsinki, and International Conference on Harmonization Guidelines for Good Clinical Practice, with approval from institutional review boards at all sites and written informed consent obtained from all patients. Clinical benefit was defined using best overall RECIST (Response Evaluation Criteria in Solid Tumors) response assessed by imaging per RECIST version 1.1, with patients categorized as having confirmed partial response (PR), stable disease (SD), or progressive disease (PD), and best percentage tumor change from baseline. SD and PD were further subdivided based on the presence (SD_Y, PD_Y) or absence (SD_N, PD_N) of active LMs. Associations with overall survival (OS) were evaluated using Kaplan–Meier curves.

**Fig. 1:**
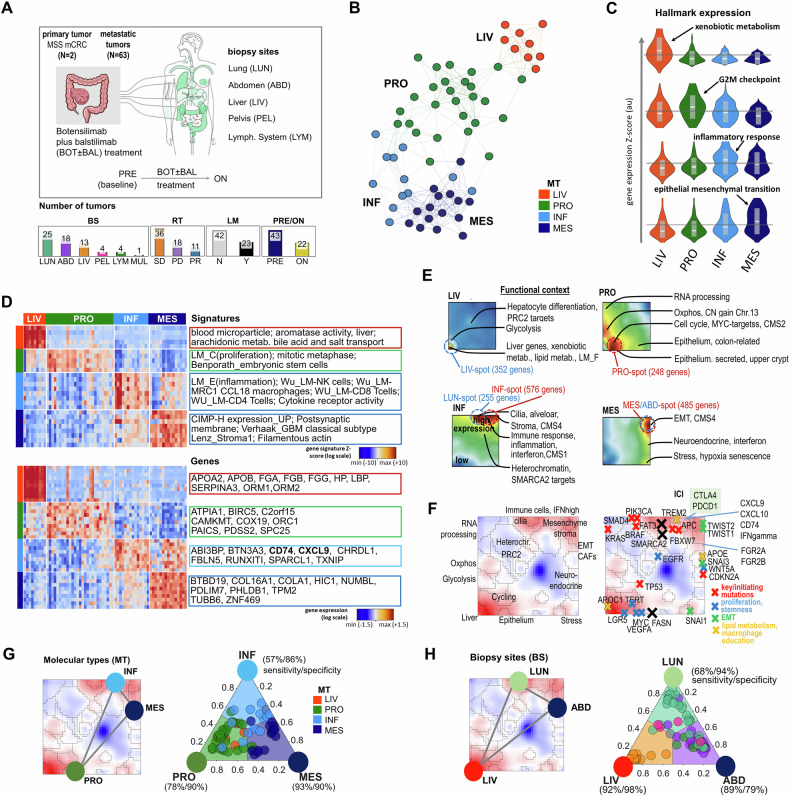
Study design, stratification, and molecular characteristics of the tumor transcriptomes. **A** C-800-01 study design: Metastases from MSS mCRC were biopsied from various sites. Tumors were stratified based on treatment response, presence of active liver metastases, and timing relative to BOT ± BAL immunotherapy (before [PRE] or after [ON]). **B** Similarity net analysis reveals four distinct MTs with unique functional themes. **C** Violin plots of selected hallmark cancer gene signatures [29] support the functional classification of MTs. **D** Differentially expressed gene sets and individual genes further characterize each MT’s functional profile. **E** Mean SOM portraits of the MTs show modules of co-expressed genes (red spots) that were upregulated in the respective MT. Overexpression analysis revealed enrichment of liver-, proliferation-, inflammation-, and stroma-related transcriptional programs in the LIV, PRO, INF, and MES MTs, respectively. **F** Summary maps illustrate the topology of characteristic functions and key genes. Gene positions remain constant across all portraits, facilitating direct comparison of expression levels. **G** MT stratification: Three core modules were selected (overview SOM, left). Gene set expression z-scores from these modules were used to construct a ternary diagram (Supplementary Figs. S9, S10). **H** Biopsy site stratification: Expression scores from three tissue modules (liver, lung, and abdominal metastases) were used to construct ternary diagrams that effectively segregate the biopsy site. The ternary diagrams separate the MT and biopsy site groups with *p* = 1.4 × 10^−11^ and 4.9 × 10^−13^, respectively (Fisher’s exact test). Sensitivity and specificity of tumors in the ternary diagrams were estimated using the respective 2×2 contingency tables. See also Supplementary Figs. S3–S10. Gene lists for different spots are provided in Supplementary Table S2. BAL balstilimab, BOT botensilimab, BS biopsy site, ID immune-depleted, IE immune-enriched, INF inflammatory MT, LIV liver-like MT, LM liver metastases, mCRC metastatic colorectal cancer, MES mesenchymal-like MT, MSS microsatellite-stable, MT molecular type, ON after BOT ± BAL treatment, PRE before BOT ± BAL treatment, PRO proliferative MT, RT response type, SOM self-organizing map, au arbitrary unit.

We analyzed bulk mRNA sequencing (RNAseq) data extracted from formalin-fixed, paraffin-embedded, tumor-containing tissue sections from 49 patients with MSS mCRC, which were obtained from diverse biopsy sites, namely liver, lung, abdomen, lymphatic system, and pelvis (*N* = 63 samples) and from the primary CRC (*N* = 2 samples) (Fig. 1A) [9, 10]. A subset of biopsies (*N* = 16) consisted of paired samples collected before (PRE) and after (ON) BOT ± BAL treatment [10]. DNA and RNA sequencing data, including mutation counts (SNPs, Indels) from whole exome sequencing (WES) and gene expression counts from RNAseq, were computed by Personalis Inc. (Fremont, CA) using their ImmunoID NeXT platform.

### RNAseq expression analysis using self-organizing maps

We applied self-organizing map (SOM) portrayal analysis to the normalized gene expression counts data [13]. The SOM machine learning–based algorithm translated the 17,250 gene expression data into 1600 metagene profiles and visualized their expression levels using a two-dimensional quadratic 40 × 40 pixel image and using a min-max color scale ranging from blue (minimum expression) to red (maximum expression) for pattern recognition (Supplementary Figs. S1, S2 [14]). SOM training was performed on centralized, quantile-normalized, log-transformed expression data with standard settings of the machine learning algorithm (quadratic grid topology, deterministic linear initialization, 250,000 iterations with decreasing learning rate, a Gaussian neighborhood function, and *Euclidean* similarity metrics [see refs. [14–16] and Supplementary Methods]), which have been proven to provide robust results in previous applications to various cancer datasets [17–21], including studies of CRC with LM [22].

We applied sample similarity analysis, which identified four molecular type (MT) clusters subsequently validated using silhouette score demonstrating distinct and cohesive groupings (Supplementary Fig. S3A, B). Co-expressed gene modules were defined using the D-module (Distance-module) method, which identified clusters of SOM pixels with high or low expression relative to the mean, as described previously [14, 16, 23]. In this study, 28 D-modules were detected; among these, we identified four core modules that served as the minimal signature required to stratify the samples into their respective MT. SOM training, module selection, differential expression analysis, function mining, and sample similarity analysis were implemented in the standardized software pipeline “oposSOM” (v. 3.0 accessed 1.02.2024) used for analysis. The entire analysis—including SOM training, class discovery (MT identification), module selection, differential expression, and functional enrichment—was performed via the oposSOM R-package (v. 3.0) [24]. Association with phenotypes was determined using correlative “phenotype portraits” and prognostic maps that relied on hazards ratios (HRs) or odds ratios (ORs), as described previously [20]. An interactive analysis platform of the SOM data is provided by the “oposSOM” browser [25] under the link http://gondwanaland.izbi.uni-leipzig.de:5978/?dataset=C800.

### Cell type deconvolution, cell type communities, and software environment

Cell-type deconvolution was performed using CIBERSORTx [26]. Cell-type communities were analyzed using the EcoTyper framework [27]. and the ESTIMATE R package [28]. For statistical analyses and plotting, we used custom scripts in Python and R (Supplementary Methods).

## Results

### Metastatic tumors arising from MSS mCRC can be organized into molecular types

We studied the transcriptomes of tissue sections of metastases of MSS mCRC patients obtained from diverse biopsy sites in the C-800-01 study (Fig. 1A) [9, 10]. A four-groups stratification of tumor samples emerged from unsupervised similarity and hierarchical clustering of SOM-transformed profiles as the most stable and functionally coherent partition in this cohort (Fig. 1B; Supplementary Figs. S1–S3; Supplementary Table S1). We named these MTs as Liver-like (LIV), Proliferative (PRO), Inflammatory (INF), and Mesenchymal-like (MES) based on subsequent gene set enrichment analysis (GSEA) (Fig. 1C) [29].

We identified differentially expressed genes and distinct transcriptional signatures for each MT. These included bile salt, lipid, and fatty acid metabolism markers, as well as liver tissue signatures in LIV; mitotic phase and proliferative stem cell signatures in PRO; immune cell, inflammatory, and cytokine-related signatures in INF; and membrane, synapse-related, and stromal signatures in MES (Fig. 1D). Specific upregulated genes included apolipoproteins (*APOA2*, *APOB*) and fibrinogen gamma/alpha/beta chain (*FGG*, *FGA*, *FGB*) in LIV; *BIRCS5*, *COX19*, and *ORC1* in PRO; *CD74* and *CXCL9* in INF; and collagen type genes (*COL1A1, COL16A1*), *HIC*, and *NUMB* in MES (Fig. 1D). Functional analyses revealed stronger similarities between LIV and PRO MTs and between INF and MES MTs, but there was also a marked heterogeneity of the tumors within each MT, indicating that the MT groups are a simplified view of more complex patterns (Supplementary Fig. S4).

Mean SOM portraits of the MTs exhibited spatial modules of up- and downregulated genes (Fig. 1E). Functional knowledge mining contextualized these patterns, revealing roles in agreement with the MT-specific signatures established above (Supplementary Figs. S5–S7). Summary maps provide an overview of molecular functions relating to different regions of the map (Fig. 1F, left). For a gene-level interpretation, we also mapped key genes into the SOM (Fig. 1F, right; Supplementary Fig. S5F). Proliferation and stemness-related genes such as *LGR5*, *MYC*, and *TERT* were located in the PRO-module; epithelial-mesenchymal transition marker genes (*TWIST1/2*, *SNAI3*) in the MES-module; and inflammatory genes and ICIs (*CD74*, *CXCL10*, *PDCD1*, and *CTLA-4*) in the INF-module. Key CRC-associated genes that frequently harbor mutations, such as *KRAS*, *SMAD4*, *FBXW7*, *PIK3CA*, and *APC* showed localization in the INF MT (Supplementary Fig. S5F).

We further visualized the MT clustering and evaluated the contribution of the biopsy site to the transcriptomic landscape by using ternary diagrams. Four core modules were identified as the primary transcriptional drivers necessary to distinguish and delineate the four distinct MTs and were denoted as LIV-, PRO-, INF-, and MES-core module, respectively (Supplementary Fig. S20). Independent component analysis revealed a continuum of molecular states of the PRO, INF, and MES MTs, while LIV tumors markedly deviated from these patterns (Supplementary Fig. S3F). We employed a ternary diagram with expression z-scores of the PRO-, INF-, and MES-core modules to visualize their relative balance (Fig. 1G, see also Supplementary Fig. S9). The distribution of PRO, INF, and MES supported the concept of the MTs as simplified stratification of a heterogeneous continuum of transcriptional states (*p* = 1.4 × 10^−11^, Fisher’s exact test) (Fig. 1G). We also generated mean SOM portraits for each biopsy site (Supplementary Fig. S8A) and similarly identified corresponding core modules. Consistent with the actual biopsy site, the core modules were annotated by oposSOM with the following enriched biological processes: liver functions for liver biopsy [22] (liver module); alveolar, lung tissue [30], and lung biopsy [31] (LUN module); and endothelial, stromal characteristics for abdominal biopsies [32] (ABD module) (Fig. 1H). Expression z-scores of the liver-, LUN-, and ABD-modules separated tumors according to their actual biopsy site (*p* = 4.9 × 10^−13^, Fisher’s exact test) with 68% (LUN) to 92% (liver) sensitivity and 79% (ABD) to 98% (liver) specificity (Fig. 1H, right), highlighting the strong contribution of biopsy site to the transcriptomic landscape (see also Supplementary Fig. S9).

This can be partly explained by healthy tissue contaminations, because tumor samples were processed without microdissection. Also, adaptations of tumor cells to their environment should be considered, e.g., the tumor-specific expression of keratin genes in lymph node metastases [33, 34] associating with cystic degeneration, the lung surfactant genes of the *SFTP*-group in lung metastases [31], and the specific mesenchymal/stroma-like transcriptomic characteristics related to epithelial adaptation of abdominal metastases [32, 35] (Supplementary Fig. S8D–F). LIV samples were exclusively derived from liver biopsies (Supplementary Fig. S8B, C). Colorectal liver metastasis expression signatures [22] revealed that the liver module is dominated by healthy liver tissue contaminations, while weaker tumor-related signals reflect predominantly proliferative and metabolic functions combined with inflammatory effects (Supplementary Fig. S8G). Sankey plots between the MT and the biopsy site indicated flow of INF tumors towards LUN biopsy site and of MES tumors towards ABD biopsy site (Supplementary Fig. S8B, C). Hence, the MT and biopsy site groups represent distinct compositions of tumor-intrinsic and tissue-specific states. The two triangulation frameworks—comprising PRO, INF, and MES, and LIV, LUN, and ABD, respectively—should be interpreted as predominantly reflecting tumor-intrinsic and tissue-specific characteristics, respectively.

### TME ecosystems of metastatic tumors enrich epithelial tumor cells, prominently fibroblast-enriched cells, and immune cells in varying compositions

**Fig. 2:**
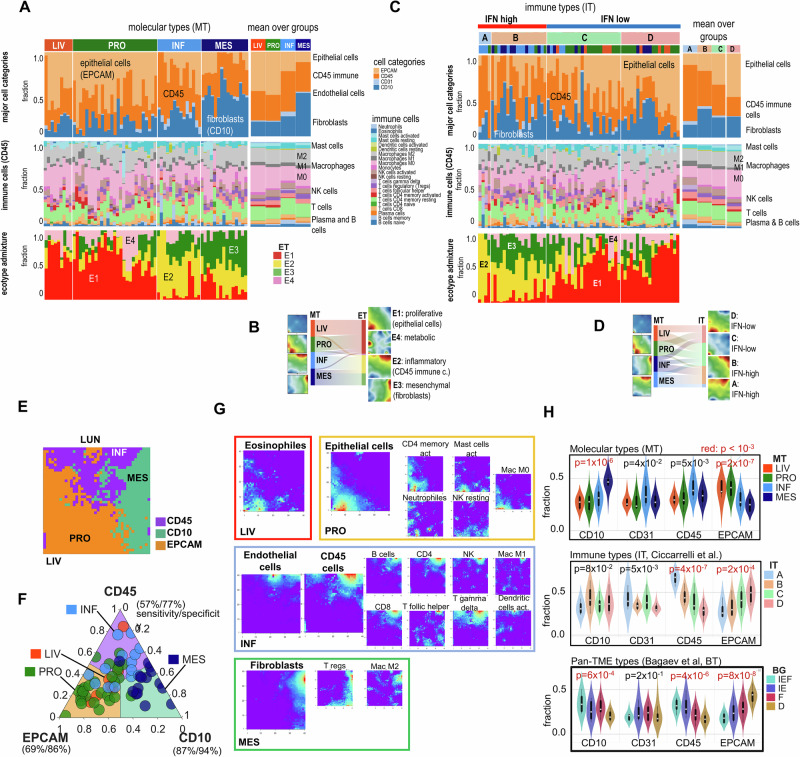
TME ecosystems and cell compositions. **A** Cell type deconvolution of the tumor bulk transcriptomes was performed using CIBERSORTx to identify multicellular communities. (i) Stacked bar plots show tumor admixture across dominant cell categories: EPCAM (epithelial tumor cells), CD45 immune cells, CD10 (primarily fibroblast-enriched), and CD31 (endothelial cells), with (ii) separate decomposition of the CD45 category into LM22 immune cells (Supplementary Fig. S13). (iii) Lower panel shows the tumor admixture into four ETs, E1-E4 (Supplementary Fig. S11). **B** Composition flow diagram between MTs and ETs reveals an almost one-to-one relationship. **C** Similar to (**A**), but with tumors re-sorted along the immunotyping scale (i-iii). **D** Flow diagram comparing compositions of MTs with ITs. **E** (i) Cell category overview map divides the SOM expression landscape into regions dominated by EPCAM, CD45, and CD10 cells. **F** Corresponding ternary composition diagram stratifying tumors by fractions of major cell categories (ii, *p* = 3.7 × 10^−10^, Fisher’s exact test, Supplementary Figs. S12, S14). **G** Cell type maps obtained by correlating expression profiles of the metagenes with cell type fractions of CIBERSORTx. Metagenes are color coded by resulting Pearson correlation coefficient (Supplementary Fig. S15). **H** Comparison of the major cell type compositions across the MTs, ITs, and TME types. Largest variance was observed for EPCAM and CD10 between the MTs, EPCAM and CD45 between ITs, and EPCAM, CD10, and CD45 between TME types (see also Supplementary Fig. S16). EPCAM epithelial tumor cells, ET ecotype, INF inflammatory MT, IT immune type, LIV liver-like MT, LM liver metastases, MES mesenchymal-like MT, MT molecular type, PRO proliferative MT, SOM self-organizing map, TME tumor microenvironment.

Cell type deconvolution revealed a decrease in epithelial tumor cells (EPCAM) and a concurrent increase in predominantly fibroblast-enriched cells (CD10, Supplementary Fig. S4I) across the molecular subtypes from LIV and PRO, through INF to MES (Fig. 2A). Immune cells (CD45) showed maximum abundance in INF (Fig. 2A, B; Supplementary Fig. S13). Ecotyping [25] identified four distinct TME ecotypes (ETs) labeled E1 to E4. The EPCAM-enriched E1 predominantly encompassed both the LIV and PRO MTs, with a minor contribution from E4, which was associated with metabolic activation. In contrast, the immune cell-enriched E2 and fibroblast-enriched E3 widely agreed with the INF and MES MTs, respectively (Fig. 2B; Supplementary Fig. S11A–F). We also applied the ESTIMATE scoring method [28] (Supplementary Fig. S11G, H).

**Fig. 3:**
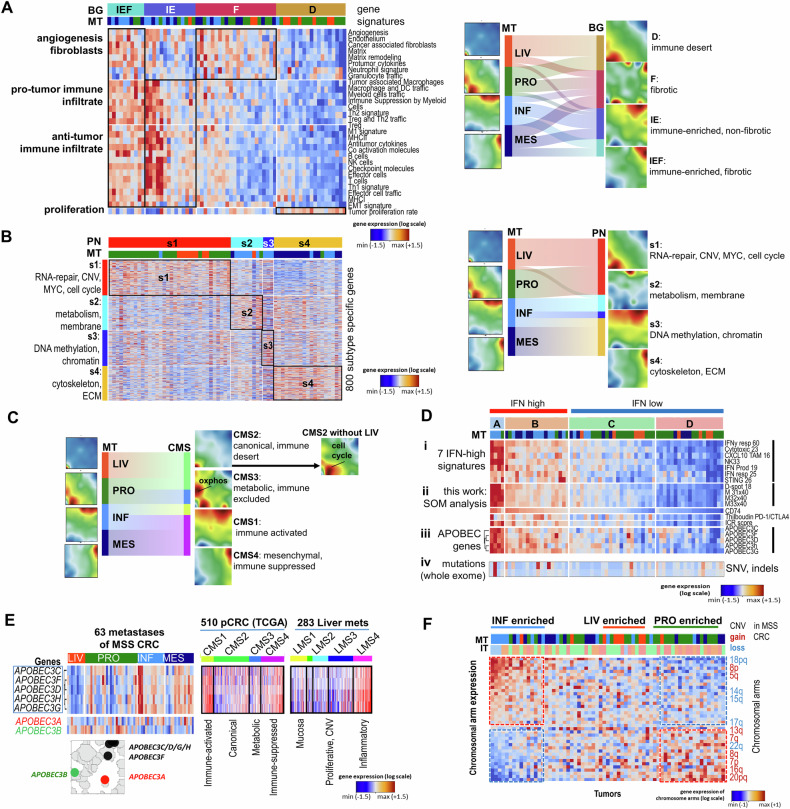
Pan-cancer classifications, immunophenotype scale, and genetic correlates. **A** Pan-TME typing divides tumors into immuno-enriched and fibrotic (IEF), immuno-enriched non-fibrotic (IE), fibrotic (F), and depleted (D) types using gene signature sets as suggested in ref. [38]. IE tumors are typically most responsive to immunotherapies, while F and D tumors are most resistant. The flow diagram shows INF and MES tumors enriched in IEF and IE groups, PRO and LIV in the D group, and F group containing a mixture of all MTs. **B** Pan-cancer metastasis typing divides tumors into four subtypes (s1-s4) based on 200 marker genes per subtype [39]. PRO and LIV MTs predominantly fit into s1, showing proliferative characteristics. INF MT tumors strongly accumulate in s2 and s3, reflecting different aspects of immune responsive and inflammatory programs. **C** Consensus molecular subtyping of CRC [40] divides tumors into CMS1-CMS4. CMS1 represents an inflammatory MSI-like state, CMS2 has proliferative characteristics, CMS3 is associated with metabolic states, and CMS4 with stromal states (Supplementary Fig. S17A). **D** (i) The interferon scale used for immunotyping [36] employs a seven-gene signature and follows CD74 expression. Similar trends are observed for genes from the interferon-spot from (ii) mCRC SOM analysis and (iii) *APOBEC3* expression, but not (iv) mutation counts of SNV and indels in tumors obtained by whole exome DNA-sequencing, which is expected to detect mutational burden. **E** Expression of *APOBEC3-C/D/F/G/H* genes across MTs, and across primary CRC and LM [22] shown for comparison, associates with inflammatory tumor states such as CMS1 and LMS4 in CRC and LM, respectively. *APOBEC-A/B* behave differently, where *APOBEC3-B* associates with proliferative functions (see also Supplementary Fig. S18). **F** Chromosomal-arm gene expression correlates with the typing and with characteristic copy number gains and losses in MSS CRC [57–59] (Supplementary Fig. S19). CMS Consensus Molecular Subtype, D depleted, F fibrotic, IE immune-enriched non-fibrotic, IEF immune-enriched and fibrotic, INF inflammatory MT, LIV liver-like MT, LM liver metastases, mCRC metastatic colorectal cancer, MES mesenchymal-like MT, MSI microsatellite-instable, MSS microsatellite-stable, MT molecular type, PRO proliferative MT, s subtype, SOM self-organizing map, TME tumor microenvironment.

An interferon score based on 7 gene expression signatures of CRC tumors was taken from ref. [36] and applied to classify the C-800-01 tumors into four immune types (ITs): interferon-high IT, clusters A and B; interferon-low IT, clusters C and D (Figs. 2C and 3D, panel i). High interferon phenotyping score correlated with high CD45 and low EPCAM abundance, as well as with the abundance of immune cell subpopulations and the sample ET (Fig. 2C). Comparison between MTs and ITs revealed correspondence between INF and partly MES with interferon-high, and between PRO and LIV with interferon-low (kappa = 0.52, *p* = 1.9 × 10^−5^, Fisher’s exact test, Fig. 2D).

For further visualization of the relative contribution of the major tissue compartments (tumor epithelial, immune, and stromal) to the MTs, we mapped the EPCAM, CD45, and CD10 cell type fractions onto the mean SOM portrait (Fig. 2E) and built a ternary diagram that separated the MTs with *p* = 3.7 × 10^−10^ (Fisher’s exact test), illustrating that the dominant transcriptional programs underlying MTs align with the major tissue compartments (Fig. 2F; Supplementary Fig. S12).

We further visualized the immune cell subsets using cell type maps (Fig. 2G; Supplementary Figs. S14–S16). Our analysis revealed distinct patterns of correlation between specific immune cell types and the previously identified MT modules. The INF-module was highly correlated with CD4+ and CD8+ T cells, follicular helper cells, gamma delta cells, NK cells, activated dendritic cells, and pro-inflammatory/antitumoral M1 macrophages [37]. In contrast, the MES-module showed correlation with fibroblasts, regulatory T cells, and anti-inflammatory/protumoral M2 macrophages. The PRO-module correlated with EPCAM tumor cells, CD4 memory T cells, activated mast cells, and naïve (M0) macrophages. Epithelial (EPCAM) and primarily fibroblast-enriched (CD10) fractions showed significant differences across MTs (*p* < 1 × 10^−3^, Kruskal–Wallis test), while differences of CD45 immune and EPCAM cell fractions showed significance across ITs. Notably, each classification system (MT and IT) was most sensitive to two of the three major cell components, underscoring the need for combined consideration of both approaches (Fig. 2H and see Supplementary Fig. S16 for differential abundance analysis between cell types). It is worth noting that cell type deconvolution, IT stratification and SOM analysis use the same transcriptomic input data.

### Pan-cancer and CRC classifications align with C-800-01 metastatic tumor types

We applied three established classification systems to our dataset: a pan-TME (“Bagaev”-types, BT) [38], a pan-metastasis classification (PMC) [39], and the Consensus Molecular Subtyping (CMS1-CMS4) [40] (Fig. 3A–C; Supplementary Figs. S17, S10). The BT classification identified immune-desert (D) and fibrotic (F) tumors in the LIV and PRO MTs, while immune-enriched tumors with (IEF) and without fibrosis (IE) were predominantly associated with the INF and MES MTs, respectively (Fig. 3A). Mean BT SOMs exhibited high expression modules similar to the MT-core modules, e.g., IE expressed the INF module, IEF displayed a superposition of MES and INF modules, F aligned with MES, and D with PRO (Fig. 3A, SOM maps). Also, PMC types showed substantial agreement with the MTs: s1 (cell cycling) aligned with LIV and PRO, s2 (metabolism, cilial endothelium) and s3 (epigenetics, interferon-high) to INF, and s4 (epithelial-mesenchymal transition) to MES (Fig. 3B). Notably, the SOM portrait of s2 strongly expressed the LUN-tissue spot, suggesting a potential bias in LUN metastasis selection.

Application of the CMS classification, originally developed for CRC, revealed further correspondences: CMS4 aligned with MES, CMS2 with PRO, and CMS3 with LIV and PRO. INF tumors with interferon-high cluster A assignment showed immunogenic CMS1 characteristics (Fig. 3C, Supplementary Fig. S17A).

In summary, we observed robust concordance between MTs, derived from our small dataset, and established classifications based on larger cohorts. This alignment reflects a shared underlying functional landscape across metastatic tumors and supports the relevance of our findings despite the limited sample size.

### The immune types are associated with *APOBEC3* expression and genomic instability

High interferon immunophenotyping score correlated with CD74 expression [36] (Fig. 3D), the INF-core module (Fig. 3D), a responder-signature of durvalumab plus tremelimumab immunotherapy against MSS mCRC [41] (Fig. 3D; Supplementary Fig. S21), and an Immunologic Constant of Rejection (ICR) score reflecting the degree of lymphocyte infiltration into the TME [42] (Fig. 3D; Supplementary Fig. S17B).

Differential gene expression analysis between the interferon-high and -low clusters revealed that expression of *APOBEC3-C/D/F/G/H* genes correlated with the interferon IT score (Fig. 3D; Supplementary Fig. S17C) (correlation coefficient 0.55–0.87, *p* < 1 × 10^−6^). High levels of *APOBEC3* gene expression were observed in the INF MT, and to a lesser degree, in the MES MT, as well as in CMS1 (immune activated) and LMS4 (inflammatory) subtypes (Fig. 3E). In contrast, *APOBEC3-A* showed no correlation with the interferon IT score, while *APOBEC3-B* was negatively correlated with it (*APOBEC3-B* correlation coefficient −0.4, *p* = 0.002). Consistently, pan-cancer analyses [43–45] describe that *APOBEC3-C/D/F/G/H* are typically co-regulated and linked to increased immune response. In contrast, *APOBEC3-B* correlates with cell proliferation, while *APOBEC3-A* exhibits inconsistent patterns [43–45].

*APOBEC3* genes encode a family of cytidine deaminases whose dysregulation can associate with localized mutational bursts, referred to as kataegis. Such mutational burden, in turn, has been associated previously with increased immunogenicity, PD-L1 expression, interferon expression, and improved responsiveness of tumors to immunotherapy [44, 46–50], including highly cytolytic, immunogenic CRC [49]. Notably, interferon can induce the expression of *APOBEC3* as part of an antiviral mutagenic defense mechanism [51, 52]. This process can, in turn, contribute to oncogenesis [53] and is concurrently associated with the generation of neoantigens [54]. Hence, the potential causal relationships among *APOBEC3* expression, tumor initiation, and the immune response are complex, possibly bidirectional, and warrant further investigation. Interestingly, the IT score strongly correlated with interferon gene activity, including APOBEC3 expression, but was not captured by conventional measures of tumor mutational burden (TMB) using WES, which relies on mutation counts in coding regions (Fig. 3D; Supplementary Fig. S18). Kataegis events are poorly identified using WES, due to limited genomic coverage and the requirement of high sequencing depth, with whole-genome sequencing remaining the preferred approach for analysis of kataegis-specific mutational signatures (e.g., SBS2/SBS13) [55, 56].

We also identified chromosomal gene expression changes suggesting an association between the tumor immune scale and genetic instability patterns in CRC (Fig. 3F; Supplementary Fig. S19). Chromosomal over- and under-expression was shown to be mostly consistent with copy number variations (CNVs) commonly observed in MSS CRC [57–60], which are largely preserved in CRC metastases [61]. We estimated mean gene expression patterns averaged over chromosome arms as proxy for CNV, suggesting association of Chr. 18p/q and Chr. 22q deletions and of Chr. 8p and Chr. 5q gains for interferon-high tumors, while Chr. 13q and Chr. 20p/q gains correlated with proliferative interferon-low tumors. Key genes and pathways affected by CNV in CRC have been shown to also impact immune surveillance, including *APOBEC3-D*, interferon signaling, and antigen presentation [62].

### Immune trajectories associate with a tumor plasticity signature gradient

**Fig. 4:**
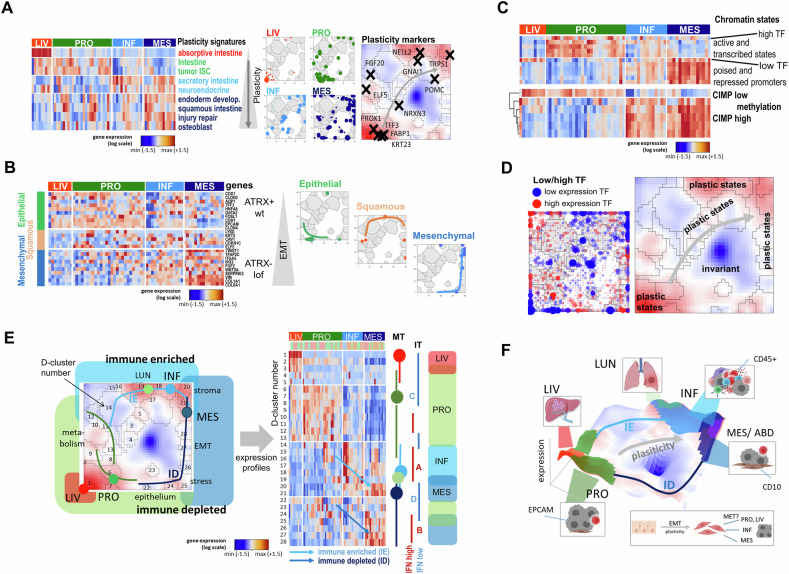
Immunophenotypes and plasticity gradient of the transcriptomic. **A** Plasticity signatures and developmental markers of CRC, as defined in ref. [63]. **B** Loss of colonic fidelity associating with *ATRX-*loss-of-function, using signatures taken from ref. [64]. **C** Epigenetic expression signatures comprising chromatin states of healthy colon [79], transcription factor (TF) activity categories related to active (high expression TF) and repressed (low expression TF) [65], and CIMP (CpG island methylator phenotype in CRC) characteristics [15]. **D** Maps of high and low TF signatures [65]. **E** The SOM landscape was segmented into 28 distinct D-modules, each exhibiting characteristic expression profiles (heatmap, right). These modules were organized into trajectory groups corresponding to the MTs (summary map, left). Colored circles denote the core D-modules representative of each MT. The summary map highlights TME regions characterized by immunophenotypes, inferred from enrichment of CD45 immune cells, CD10 primarily fibroblast-enriched cells, and EPCAM tumor epithelial cells. INF- and MES-related trajectories were classified as immune-enriched (IE) and immune-depleted (ID), respectively. The module associated with lung metastases is marked by a light green circle. IE and ID characteristics are indicated by arrows highlighting the observed continuum of transcriptomic profiles that goes beyond MT classifications. **F** Overview map of the tumor transcriptome and the associated TME and biopsy site. These patterns delineate immune-depleted, mesenchymal fibrotic, and immune-enriched TME ecosystems, aligned along a plasticity gradient of the tumor cells from epithelial to mesenchymal states. CIMP CpG island methylator phenotype, CRC colorectal cancer, EPCAM epithelial tumor cells, ID immune-depleted, IE immune-enriched, INF inflammatory MT, IT immune type, LIV liver-like MT, MES mesenchymal-like MT, MT molecular type, PRO proliferative MT, SOM self-organizing map, TF transcription factor, TME tumor microenvironment.

A recent study demonstrated that loss of intestinal epithelial cell identities, accompanied by increased cellular plasticity, transcriptional reprogramming, and non-canonical differentiation into squamous and neuroendocrine-like states, drives CRC metastasis [63]. Gene expression signatures of plasticity and developmental markers of CRC taken from [63] were differentially enriched in the four MTs: absorptive intestine signature predominated in LIV and PRO, secretory intestine and neuroendocrine signatures in INF, and squamous intestine signature in MES (Fig. 4A heatmap). However, we observed heterogeneity both at the sample level (Fig. 4A heatmap) and in the distribution of single genes comprising these plasticity signatures across the SOM map (Fig. 4A SOM maps), pointing to a plasticity gradient or continuum rather than a definite separation. A similar plasticity gradient was observed with gene expression signatures associated with loss of function for chromatin-remodeling machinery, including *ATRX*, a key regulator of colonic lineage fidelity and metastasis [64] (Fig. 4B). *ATRX* loss of function promotes tumor invasion and metastasis, concomitant with a loss of colonic epithelial identity and the emergence of highly plastic mesenchymal and squamous-like cell states [64]. The plasticity gradient aligned with the expression patterns of gene signatures representing active (CpG island methylator phenotype; CIMP low) and repressed (CIMP high) promoter states in the colon epithelium (Fig. 4C). It also corresponded to the distribution of low- and high-expression transcription factor (TF) genes implicated in cell fate transitions driven by epigenetic reprogramming [65] (Fig. 4C, D).

SOM self-organization properties arranged modules with similar expression profiles closely together, thus providing continuums of heterogeneous transcriptional profiles, or “trajectories” (Fig. 4E; Supplementary Fig. S5, see also refs. [66, 67]). We identified two such trajectories overlapping with the INF- and MES-core modules, which showed immune-enriched (IE, light blue curves) and immune-depleted (ID, dark blue curves) characteristics, respectively, while PRO-related modules were designated as immune-desert (green) and LIV-related modules as immuno-suppressed states (red). The heatmap of the expression modules D1-D28 expresses the differences between IE and ID trajectories, namely that INF-type tumors show elevated activity across the IE modules, while MES-type tumors exhibit decreased immune characteristics (Fig. 4E, right part, Supplementary Figs. S5, S7, Supplementary Table S2).

In summary, the transcriptomic landscape reveals distinct TME immunophenotypes—ID or IE—and defines a tumor cellular plasticity gradient (Fig. 4F). The *APOBEC3*/interferon-high region overlaps with signatures of increased plasticity and loss of colonic fidelity, including the ATRX loss-of-function signature (Fig. 4A, B). We also observed concordant enrichment of expression-based CIMP-high promoter-state signatures toward INF/MES compared with PRO/LIV (Fig. 4C). Finally, interferon-high/*APOBEC3*-high tumors show patterns consistent with reduced Chr18p/q and Chr22q and increased Chr5q and Chr8p expression, whereas interferon-low/proliferative tumors show increased Chr13q and Chr20p/q expression (Fig. 3F; Supplementary Fig. S19). These findings support the hypothesis that tumor heterogeneity is shaped by both the developmental state of epithelial tumor cells and their dynamic interactions with the TME and its varying composition, although the ATRX loss, CIMP status, and copy-number changes were inferred from expression-based signatures rather than directly measured by DNA/methylation assays and thus require orthogonal validation; and the bulk nature of the data did not allow for evaluation of the potential confounding effect of the proportion of stromal compartment in the analyzed samples.

### BOT ± BAL treatment shifts tumors towards immune-enriched states

**Fig. 5:**
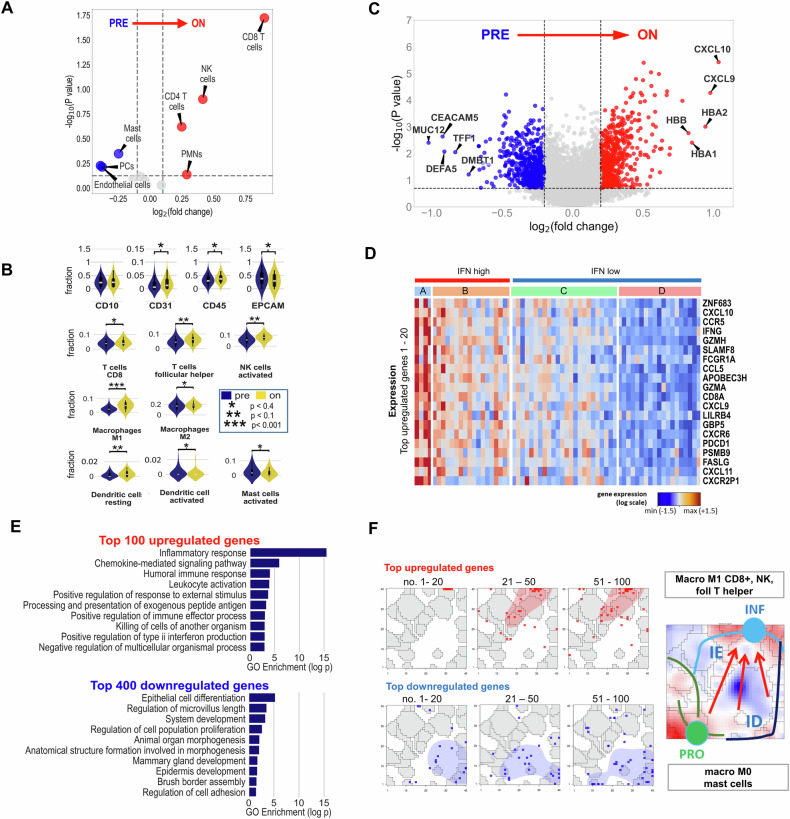
Immunotherapy enhances TME immunogenicity. **A** Volcano plot illustrating differential cell type abundance reveals increased content of CD8+, CD4+ and follicular helper T cells, M1 macrophages, and NK cells in the TME of treated tumors. **B** Violin plots comparing predicted abundances of selected immune cells in pre-treatment (PRE) and on-treatment (ON) settings of BOT ± BAL immunotherapy. **C** Volcano plot illustrating differential gene expression between PRE and ON with BOT ± BAL immunotherapy. Gene lists are provided in Supplementary File 3: Supplementary Table S2. **D** Top upregulated genes accumulate in interferon-high states along the IT scale. **E** Gene set analysis shows upregulated genes enriched in immunogenic themes, while downregulated pathways include epithelial development. **F** Mapping of DEGs into the SOM shows that upregulated genes (ranked by fold change) are predominantly enriched in the INF-module and along the IE-trajectory, while downregulated genes are concentrated along the ID-trajectory. These patterns suggest a directional shift in gene activity towards a more immunogenic TME from the ID- to the IE-trajectory, as depicted by arrows on the adjacent trajectory map. BAL balstilimab, BOT botensilimab, ID immune-depleted, IE immune-enriched, INF inflammatory MT, IT immune type, MT molecular type, ON after BOT ± BAL treatment, PRE before BOT ± BAL treatment, SOM self-organizing map, TME tumor microenvironment.

We analyzed data from patients with paired biopsies collected before (PRE) and after (ON) BOT ± BAL treatment. Comparison of cell compositions revealed treatment-induced infiltration of CD45 immune cells into the TME (Fig. 5A, B). This infiltration included CD8+ and CD4+ T cells, anti-tumoral M1 macrophages, NK cells, and follicular helper T cells. Concurrently, we observed depletion of epithelial tumor cells and of naïve (M0) macrophages, consistent with prior findings [10].

Differential gene expression analysis identified up-regulation of several immune-related genes (Fig. 5C, D; Supplementary Table S3). These included *ZNF683*, a marker of non-exhausted T cells predictive of anti–*PD-1* response [68]; *CXCL9/10*, critical for macrophage-mediated anti-tumor immunity following immune checkpoint blockade [69]; *interferon-γ*, an inducer of M1 macrophage polarization [70]; *GZMA*, *GZMB*, and *GZMH*, which promote cytotoxic activity [71]; *CCL5*, known to enhance M1 polarization in the context of immunotherapy [72]; and *APOBEC3-G/H*, potentially related to DNA methylation changes associating with the TME [73].

Pathway analysis of the up-regulated genes showed enrichment in interleukin production, chemokine and interferon signaling, and NK activation (Fig. 5E) pathways that are known to be associated with response to immunotherapy in CRC [74]. Conversely, pathways enriched in the down-regulated genes were associated with epithelial, developmental, and stress states. The top up-regulated genes clustered at the top right of the SOM, around the INF spot, while the top down-regulated genes clustered more broadly in the bottom right part of the SOM, along stress, hypoxia, and senescence modules, among others (Fig. 5F) in qualitative agreement with an independent study of anti–*PD-1* and anti–*CTLA-4* treatment of CRC [41] (Supplementary Fig. S21). Collectively, these findings indicate that BOT ± BAL treatment shifted the tumors towards immune hot states, which are expected to respond better to ICIs. We do not interpret the paired-biopsy findings as a formal re-assignment of a tumor to a different MT, nor as evidence of stable lineage/state conversion; rather, the paired analysis is intended to capture acute treatment-associated shifts in immune-related programs within the existing transcriptomic landscape.

### INF and MES tumors show a better treatment response and prognosis compared with PRO and LIV tumors

**Fig. 6:**
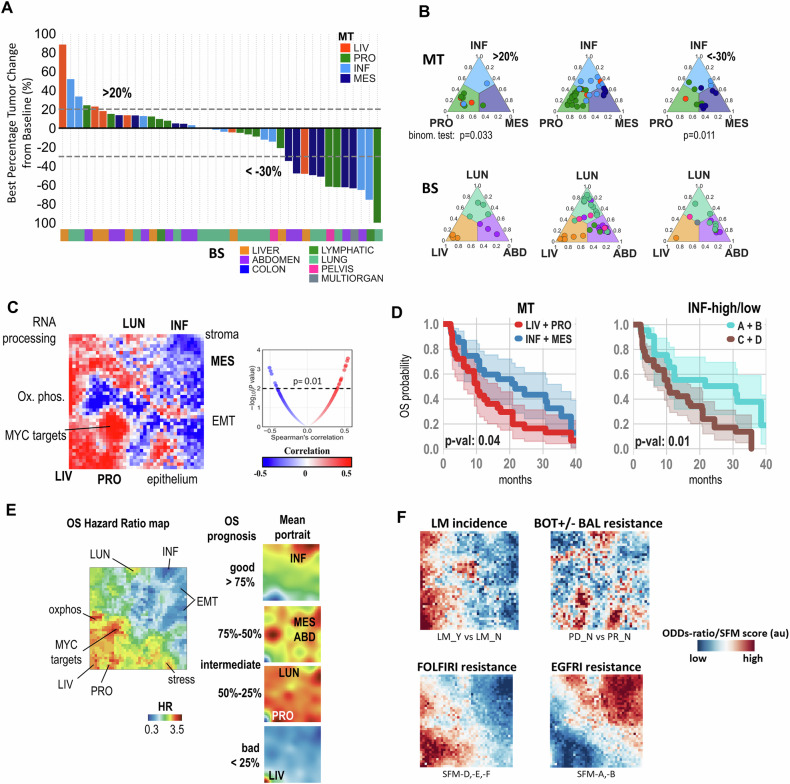
Response of MSS mCRC tumors to BOT ± BAL immunotherapy. **A** Tumors ranked by best percentage tumor change from baseline during immunotherapy. **B** The ternary diagrams reveal accumulation of tumors in the PRO-corner for growing tumors with best percentage change from baseline >20% (*p* < 0.033, binomial test) and in the INF and MES corners for <30% (*p* = 0.011). **C** Increase in tumor size positively correlates with regions of the SOM landscape enriched for PRO and LIV metagenes (red pixels) and negatively correlates with regions associated with INF and MES subtypes (blue, Supplementary Fig. S21). **D** OS curves suggest better prognosis for interferon-high compared to interferon-low and of INF + MES MTs compared with PRO + LIV MTs (Supplementary Fig. S23). **E** The OS map displays the HRs associated with metagene expression profiles. Minimum HR values (blue) are found near the INF-core module. The mean SOM portrait corresponds to the HR quartiles, revealing associations with INF-, MES-, PRO-, and LIV-modules, reflecting a continuum from favorable to poor prognosis. **F** Response maps visualize the OR of various clinical outcomes. These include the incidence of liver metastases (LM) comparing PD_Y and SD_Y versus PD_N and SD_N tumors, resistance against BOT ± BAL immunotherapy comparing PD_N versus PR_N tumors, and resistance against FOLFIRI chemotherapy and EGFR inhibition (EGFRi) using scores derived from ref. [75] for comparison (Supplementary Fig. S24). BAL balstilimab, BOT botensilimab, EGFRi epidermal growth factor receptor inhibitor, HR hazard ratio, INF inflammatory MT, LIV liver-like MT, LM liver metastases, mCRC metastatic colorectal cancer, MES mesenchymal-like MT, MSS microsatellite-stable, MT molecular type, N no, OR odds ratio, OS overall survival, PD progressive disease, PR partial response, PRO proliferative MT, SD stable disease, SOM self-organizing map, Y yes, au arbitrary unit.

We performed a correlative analysis of the tumors’ molecular profiles with clinical response and OS (Fig. 6). Tumors exhibiting a >30% reduction in size were predominantly in the INF and MES MTs (Fig. 6A) and accumulated in the respective corners of the ternary diagrams (*p* = 0.011, binomial test), while patients with growing tumors >20% were enriched in the PRO corner (*p* = 0.033, Fig. 6B). Gene expression profiles in and near the PRO and LIV modules positively correlated with changes in tumor size, while expression in the MES and INF modules negatively correlated with it (*p* < 0.05, Fig. 6C; Supplementary Fig. S22). Given this finding and the small number of patients in each MT, we grouped MES with INF and LIV with PRO for the OS analysis. We observed better OS for interferon-high ITs compared to interferon-low ITs (*p* = 0.01, log-rank test) and for MES and INF MTs compared to LIV and PRO MTs (*p* = 0.04, Fig. 6D; Supplementary Fig. S23; Supplementary Table S4). Gene expression in the INF and MES modules was associated with better OS (HR < 1, relative to the mean OS), while expression in the LIV and PRO modules was associated with worse OS (HR > 1), demonstrating the favorable impact of immunogenic phenotypes and unfavorable impact of liver metastasis and proliferative states (Fig. 6E; Supplementary Fig. S24C). Beyond these cohort-level enrichments, responses to BOT ± BAL were not exclusive to INF/MES, as a subset of PRO-classified tumors did respond, including one patient with a particularly deep response (Fig. 6A).

We visualized the OR of resistance to BOT ± BAL across the SOM in patients without active LM and with active LM both showing increased resistance/incidence in the left part of the map referring to LIV and PRO MTs (Fig. 6F). The incidence of active LM was also associated with metabolic activity and RNA processing. For comparison, we calculated a prognostic map using gene expression signatures previously associated with CRC resistance to chemotherapy (FOLFIRI) and for epidermal growth factor receptor inhibitors (EGFRi) taken as external transcriptomic predictors from earlier-line cohorts in [75] (Fig. 6F; Supplementary Fig. S24), which suggested a similar (chemo resistance associated with LIV and PRO tumors) and an opposite (EGFRi sensitivity associated with highly proliferative LIV and PRO tumors) pattern to that seen for immunotherapy, respectively (Supplementary Fig. S24C). With the limitation that these predictions were only based on transcriptomic signatures defined from external independent cohorts, these results could be rationalized by the anti-proliferative action of EGFRi and the association of FOLFIRI with immune response [75, 76]. In a broader context, this underlines the emerging importance of combined therapy options [77, 78].

## Discussion and conclusions

Taken together, these data and our analysis provide a clinically meaningful framework for understanding why a subset of heavily pretreated, chemo-refractory MSS mCRC patients derive durable benefit from BOT ± BAL. By integrating SOM-based transcriptional mapping with multiple external classification systems, we consistently identified distinct molecular types: an inflammatory, immune-enriched, interferon-high, *APOBEC3*-high type aligning with the inflamed CMS1-like CRC subtype; a mesenchymal, immune-enriched, fibrotic TME type resembling CMS4 tumors; and proliferative and liver-like types that are epithelial, metabolic, and immune-depleted, with the LIV MT transcriptome dominated by features resembling healthy liver tissue (Figs. 1–3). These four MTs are derived from unsupervised clustering that summarize dominant transcriptional programs and reflect interpretable states along a continuum rather than rigid categories. Clinically, patients whose baseline tumors fell into INF/MES or the interferon-high IT had greater depth of tumor shrinkage and superior OS after BOT ± BAL vs those harboring PRO/LIV, immune cold lesions, despite uniformly low TMB and prior failure of standard chemotherapy and biologics (Fig. 6).

The identification of an immunophenotype axis marked by progressive expression of *CD74*, *interferon-γ signature*, and *APOBEC3* genes was a notable finding. The longitudinal analysis of paired biopsies showed that BOT ± BAL treatment can shift some lesions along a plasticity gradient toward immune-enriched states, with increases in CD8 and CD4 T cells, NK cells, M1 macrophages, and interferon inducible chemokines, and concomitant down modulation of epithelial/developmental stress programs (Figs. 4, 5 and ref. [10]), supporting a model in which BOT ± BAL both selects for and actively promotes immunophenotypes in MSS mCRC. The tight linkage between interferon signaling, *APOBEC3* expression, and favorable outcomes, in the absence of a parallel association with exome-wide mutation counts, suggests that focal hypermutation and related “quality” features of neoantigens may contribute to clinical benefit beyond conventional measures of TMB, although this remains a hypothesis that will require direct DNA-level validation in larger cohorts. Notably, *APOBEC3* expression correlated with the immunophenotype gradient independent of overall mutational burden, suggesting that localized hypermutation contributes to immune recognition and positioning *APOBEC3* as a promising candidate biomarker and potential target for cancer immunotherapy due to its mutagenic properties as hypothesized previously [53]. Clinically, these findings argue that transcriptomic and microenvironment features, particularly interferon-high INF/MES TMEs and non-liver metastatic sites, are more informative than global mutation burden for anticipating benefit from Fc-enhanced *CTLA-4*–based immunotherapy in MSS mCRC, and they provide a conceptual map for rational combinations aimed at converting PRO/LIV, immune-cold metastases into more treatment-sensitive immunophenotypes.

While our data identify key molecular mechanisms that underlie immunotherapy responsiveness in resistant MSS mCRC, several limitations should be considered. The modest sample size restricts statistical power and generalizability of effect estimates, and the use of bulk transcriptomic profiling may obscure contributions from rare, spatially localized cell types. Additionally, the mechanistic role of *APOBEC3* and the link between plasticity axes, genetic instability (CNV, focal hypermutation), and immune activation remains to be established through genomic (e.g., kataegis clustering or mutational signatures SBS2/SBS13 analysis using WGS) and functional studies and validated in larger cohorts. Protein-level verification of the RNAseq results was not feasible due to limited clinical tissue availability from the tumors. Future studies incorporating orthogonal approaches such as single-cell or spatial transcriptomics, protein analysis, and DNA-level longitudinal analyses of mutational signatures/clones would be valuable for validation of our findings. Future studies with higher-purity tumor material and/or single-cell or spatial profiling will be needed to resolve tumor-intrinsic programs in liver lesions more definitively.

Overall, this work advances our understanding of the molecular architecture shaping MSS mCRC metastasis and response to ICIs. The identification of tumor plasticity gradients, *APOBEC3*-associated immunogenicity, and treatment-induced immune reprogramming provides mechanistic insights into both intrinsic and acquired resistance to checkpoint blockade. Importantly, these findings highlight actionable molecular signatures and suggest new avenues for biomarker-driven patient stratification and rational therapeutic combinations that may improve outcomes for patients with MSS mCRC.

## Supplementary information

Supplementary File 1

Supplementary File 2

Supplmentary File 3

## Data Availability

De-identified individual participant clinical data and WES and bulk RNA-seq data that underlie the results reported in this article are available for transfer upon request for academic use and within the limitations of the provided informed consent. Interested investigators can obtain and certify the data transfer agreement and submit requests to AGENUS Inc. (D. Chand, see also Supplementary Methods).
