# Supplementary material for: Transcriptomic profiling identifies immunotherapy-responsive phenotypes in microsatellite-stable metastatic colorectal cancer
Source: Oncogene. 2026 Jun 16;45(29):2932–44. doi: 10.1038/s41388-026-03861-2 (PMC13364656; doi:10.1038/s41388-026-03861-2)
Supplement: Supplementary file 1 [file 41388_2026_3861_MOESM1_ESM.docx]

**Supplementary Material: Supplementary Methods, Supplementary Table S1 and S4, and Supplementary Figures S1 – S24**

**Transcriptomic Profiling Identifies Immunotherapy-Responsive Phenotypes in Microsatellite-Stable Metastatic Colorectal Cancer**

Tomas Konecny^1,2#^, Nate Zadirako^1,3#^, Arpine Grigoryan^1^, Melina Tamazyan^1,3^, Sveta Mnatsakanyan^1,3^, Luiza Stepanyan^1^, Henry Loeffler-Wirth^2^, Sean Bourdelais^4^, Gabriel Mednick^4^, Chloe Delepine^4^, Dhan Chand^4,§^, and Hans Binder^1,2,3,§^

1 Armenian Bioinformatics Institute (ABI), 7 Hasratyan Str., Yerevan 0014, Armenia

2 Interdisciplinary Centre for Bioinformatics (IZBI), Universität Leipzig, Härtelstr. 16 – 18, 04107 Leipzig, Germany

3 Institute of Molecular Biology of the National Academy of Sciences of the Republic of Armenia (IMB), 7 Hasratyan Str., Yerevan 0014, Armenia

4 Agenus Inc., 3 Forbes Road, Lexington, MA 02421-7305, USA

# contributed equally

§ shared senior authorship

**Supplementary Methods**

**Study design, patient data, treatment, and response evaluation**

***Study design*:** The C-800-01 study is an open-label, phase 1b, multicenter study evaluating the safety, tolerability, and efficacy of BOT±BAL (NCT03860272). The subset of patients with MSS metastatic colorectal cancer (mCRC), which had WES/RNAseq data available were enrolled and treated between April 23, 2020, and January 12, 2023, at 11 sites across the United States. The full study design, dose escalation, and results from the MSS mCRC and sarcoma cohorts have been previously reported [1-3].

Eligible patients were age 18 years and older with measurable disease per Response Evaluation Criteria in Solid Tumors (RECIST) version 1.1 and had an Eastern Cooperative Oncology Group (ECOG) performance status (PS) of 0 or 1 with adequate end-organ function. Previous I-O was permitted. The trial was conducted in compliance with the Declaration of Helsinki and International Conference on Harmonisation Guidelines for Good Clinical Practice and was approved by the institutional review board at each institution, with all patients providing written informed consent.

***Procedures***: Botensilimab (BOT) was administered intravenously (IV) once every 3 weeks or once every 6 weeks (Q6W) at dose levels of 0.1-3 mg/kg for up to 2 years ± balstilimab (BAL), administered IV once every 2 weeks (Q2W) at a dose level of 3 mg/kg, during the dose escalation, both up to 2 years. Patients enrolled during the dose-expansion portion of the trial received 1 or 2 mg/kg of BOT (once Q6W) in combination with BAL 3 mg/kg (once Q2W), both for up to 2 years. Patients were permitted to crossover from BOT monotherapy to the combination and could be treated beyond progression at the discretion of the managing medical team. Of the 49 patients included in this correlative study, 7 received BOT monotherapy and 42 were treated with BOT+BAL combination, including 2 who crossed over after initial monotherapy treatment. The C-800-01 trial is closed to enrolment.

***Endpoints:*** The primary endpoint was the assessment of safety and toxicity via the occurrence of dose-limiting toxicities (DLTs) during dose escalation. As previously reported [2], no DLTs occurred during this period in the MSS mCRC cohort and a maximum tolerated dose was not reached (NR). Secondary endpoints included the assessment of severity and duration of treatment-emergent adverse events (TEAEs) and laboratory abnormalities, according to the National Cancer Institute's Common Terminology Criteria for Adverse Events (NCI-CTCAE version 5.0). Secondary efficacy endpoints were Best Overall RECIST Response (BORR), duration of response (DOR), disease control rate (DCR; defined as a complete response [CR], partial response [PR], or stable disease [SD] of ≥6 weeks), and progression-free survival (PFS), all per RECIST v1.1 based on investigator assessment. OS was an exploratory endpoint. Clinical benefit rate (CBR) was also reported and defined as a best response of CR, PR, or SD for ≥24 weeks. Imaging was performed once every 6 weeks (±3 days) from the first treatment.

**Whole exome sequencing and bulk RNAseq**

Genomic DNA and RNA from formalin-fixed, paraffin-embedded (FFPE), tumor-containing tissue sections and matched blood samples were isolated and subjected to whole-exome paired-end sequencing and RNA sequencing (RNA-seq) using the Personalis ImmunoID NeXT platform. All patients provided written informed consent. DNA/RNA extraction, library preparation, NovaSeq sequencing, and data analysis were performed at Personalis, demonstrating accuracy and precision of their experimental protocols, with a sensitivity of 99% for single-nucleotide variants (3% false-positive rate) and 94% for indels (3% false-positive rate). The coverage was 150× for germline control blood samples and 300× for tumor samples.

The Personalis pipeline integrates open source, commercial, and proprietary tools to detect and report somatic single nucleotide variants (SNVs) and small (<50 bp) insertions and deletions (indels). The Personalis pipeline produced a set of somatic SNV and indel calls based on i) alignment metrics, such as sequence coverage and read quality, ii) positional features, such as proximity to a gap region, and iii) likelihood of presence in normal tissue. Gene expression counts in units of transcripts per million (tpm) were reported by Personalis as per their analysis pipeline.

**Downstream bioinformatics analysis**

***Data portrayal using self-organizing maps machine learning:*** Gene expression data were transformed into logarithmic scale, quantile normalized, then centralized and clustered using self-organizing map (SOM) portrayal machine learning using the software "oposSOM" (version 3 available from [https://github.com/hloefflerwirth/oposSOM accessed 1.02.2024](https://github.com/hloefflerwirth/oposSOM%20accessed%201.02.2024) and updated on January 2026) and default parameter settings [4].

It transforms the N × M expression matrix (N=17,250, number of genes; M=65, number of tumors) into a reduced-dimensionality K × M matrix, where K denotes the number of so-called metagenes. In systematic methodological studies we have shown that SOMs with sizes between K = 40 × 40 and 60 × 60 metagenes yield robust results in terms of detecting modules of coregulated genes in typical applications involving large-scale transcriptional datasets comprising tens of thousands of genes [5]. In the present study, we employed a two-dimensional SOM grid of size K = 40 × 40 = 1,600 metagenes. Metagenes are arranged in a quadratic grid topology and initialized by linear combinations of the first two eigenvectors of the gene expression matrix with two-dimensional grid-coordinates as coefficients (deterministic linear initialization). Then, metagenes are successively adapted to the single gene training profiles during more than 200.000 iterations (12 epochs) with decreasing learning rate and Gaussian neighborhood radius. In each iteration, best-matching metagene of a training gene is determined by minimal *Euclidean* distance, followed by adaption of all metagenes whereby the adaption decreases with increasing distance to the best-matching metagene [6]. These parameters have been proven to provide robust results in previous applications to various cancer datasets [16-20], including studies of CRC liver metastases [21]. Modules of co-expressed genes were identified using the D-module method as described previously [14, 15, 22].

Each tumor expression portrait is represented by the resulting K metagene expression values, arranged according to the SOM grid and visualized using a min-max color scale ranging from blue (minimum expression) to red (maximum expression) (Figure S1). The resulting color patterns form smooth textures that portrait the gene expression fingerprints of individual tumors. Mean expression portraits for tumor groups were computed by averaging the expression values of each metagene across all tumors within a group and visualized accordingly.

Metagenes with similar expression profiles cluster together due to the self-organizing properties of the SOM, forming distinct "spot-like" regions of over- and under-expression in the portraits. Each spot corresponds to a module of mutually correlated genes. These modules were defined by D-clustering using a distance-based criterion based on the *Euclidean* distance between neighboring metagenes, whereby metagenes with maximal mutual distances often form closed, halo-like boundaries surrounding the spots [7]. Importantly, the detection of signature modules is performed in an unsupervised manner, without the need for prior specification of prototypes or the number of clusters. Lists of genes included in each of the spot modules provide the associated functional context using gene set analysis [8].

All described functions (SOM training, spot-module selection, differential expression analysis, function mining, and sample similarity analysis) were performed using oposSOM [9]. Association of metagene expression of the SOM with survival data was visualized as prognostic maps using hazard or odds ratios (relative to the mean prognosis of all tumors and mean expression of the metagenes across all samples) as described previously [10, 11].

***Cell type deconvolution and ecotyping:*** Cell-type deconvolution of bulk RNAseq data was performed using CIBERSORTx [12, 13] that emerged with strong performance detecting abundances of cell types within both healthy samples and tumor tissues [14]. CIBERSORTx is among the top benchmarked cell deconvolution methods (see, e.g., [14-16]). CIBERSORTx detects a large set of cell types and, as a reference-based method, computes proportions also comparable across cell types [14].

First, the program separates immune cells (expressing CD45), endothelial cells (CD31), fibroblasts (CD10), and epithelial/tumor cells (EPCAM) using an experimentally derived signature matrix obtained from FACS-purified tumor cells labelled with the surface markers CD45, CD31, CD10, and EPCAM, respectively, and assigned to immune cells, endothelial cells, fibroblasts, and epithelial tumor cells, respectively [13]. We use the marker abbreviations as proxy names for the respective cell types throughout the manuscript. Then, the CIBERSORTx program divides the immune compartment into the 22 immune cell types represented by the LM22 signature matrix originally published with CIBERSORT [12]. The EcoTyper machine learning framework is then used for large-scale identification of cell states and cellular ecosystems [17]. The ESTIMATE R-package was used as an additional method to estimate tumor cell, immune cell and stromal cell contents of the tumors [18].

***Cell-type (abundance) maps:*** Maps associating metagene expression of the SOM with cell type fractions of the CIBERSORTx were obtained by computing the dot product of each metagene expression vector with a selected cell fraction vector across all tumors, thus providing a cell fraction weighted expression value for each metagene. Here, the respective metagene expression in each tumor is multiplied by the respective cell type fraction. The resulting value is high when high expression is paralleled by high cell fractions throughout the tumors and low when either one or both factors are small. Mapping and visualizing these values into the SOM-grid with color-coding from red (high value) to blue (zero, low) provides the abundance map for the cell type chosen.

For statistical evaluation, we also generated correlation maps plotting the Spearman’s correlation coefficient for the correlations between each metagene expression vector and the respective cell type fraction vector.

***Chromosomal expression and prediction of Copy Number Variants (CNV)*:** Chromosomal expression was assessed for all chromosomes using a sliding window of 50 genes along the entire chromosome. The average expression of all genes from each chromosome arms of interest (harboring known CNV in CRC) was used as a proxy for CNVs.

***Statistical analysis and software environment:*** For statistical analyses and plotting, we used custom scripts in Python (version 3.12.11 or 3.14.0) using built-in packages as well as third-party packages, like lifelines (version 0.30.0), matplotlib (version 3.10.7), numpy (version 2.1.3 or 2.3.4), pandas (version 2.2.3 or 2.3.3), plotly (version 6.3.0), rpy2 (version 3.5.16), scipy (version 1.15.3 or 1.16.3), seaborn (version 0.13.2), and scikit-learn (version 1.6.1). Linear relationships between variables were evaluated with Pearson correlation tests, and correlation coefficients and p-values were reported. Proportion distribution of the samples in the corners of the ternary diagrams was assessed with binomial test. Kruskal-Wallis test was used for comparisons across molecular types (MTs).

***Survival analysis***: OS analysis was done using Kaplan–Meier estimate with the clinical data cutoff of March 13, 2025, and log-rank test hazard ratios and p-values were reported. OS was censored at the last contact date unless a death was reported for a patient prior to the data cutoff date. The log-rank test was used to compare OS between groups.

**Data availability**

De-identified individual participant clinical data and WES and bulk RNA-seq data that underlie the results reported in this article are available for transfer upon request for academic use and within the limitations of the provided informed consent. Interested investigators can obtain and certify the data transfer agreement and submit requests to AGENUS Inc. (D. Chand). Investigators who consent to the terms of the data transfer agreement, including, but not limited to, the use of these data only for research purposes, and to protect the confidentiality of the data and limit the possibility of identification of patients in any way whatsoever for the duration of the agreement, will be granted access. Data will be available for request for a period of 2 years after the completion of the C-800-01 study. Requests will be evaluated on a case-by-case basis for a period of at most 2 weeks before receipt of a response.

An interactive analysis platform of the data is provided by the "oposSOM" browser [19] under the link <http://gondwanaland.izbi.uni-leipzig.de:5978/?dataset=C800>.

**Supplementary Table S1**

**Table S1: Patient characteristics of the molecular types (MTs). (A)** **Demographic distribution of selected characteristics. (B)** **Significance (p-values) of pairwise comparisons.**

Table S1A: Demographic distribution of selected characteristics.

| Patient characteristic | INF  (n=12) | LIV  (n=7) | MES (n=12) | PRO (n=18) | all  (n=49) |
| --- | --- | --- | --- | --- | --- |
| Age, years |  |  |  |  |  |
| Median (range) | 54.5  (36-81) | 54  (36-73) | 60.5  (46-72) | 53.5  (25-82) | 56  (25-82) |
| Sex |  |  |  |  |  |
| Female, No. (%) | 6 (50%) | 4 (57%) | 5 (42%) | 11 (61%) | 26 (53%) |
| Male, No. (%) | 6 (50%) | 3 (43%) | 7 (58%) | 7 (39%) | 23 (47%) |
| ECOG PS |  |  |  |  |  |
| 0, No. (%) | 3 (25%) | 3 (43%) | 3 (25%) | 8 (44%) | 17 (35%) |
| 1, No. (%) | 9 (75%) | 4 (57%) | 9 (75%) | 10 (56%) | 32 (65%) |
| BMI |  |  |  |  |  |
| Median (range) | 27 (18-36) | 26 (20-38) | 31 (22-42) | 23 (17-33) | 26 (17-42) |
| Prior lines of therapy |  |  |  |  |  |
| Median (range) | 4 (1-9) | 4 (3-8) | 5 (1-7) | 4 (2-9) | 4 (1-9) |
| ≥3, No. (%) | 8 (67%) | 7 (100%) | 10 (83%) | 16 (89%) | 41 (84%) |
| Prior anti–*PD-(L)1/CTLA-4* |  |  |  |  |  |
| Yes, No. (%) | 5 (42%) | 2 (29%) | 0 (0%) | 4 (22%) | 11 (22%) |
| No, No. (%) | 7 (58%) | 5 (71%) | 12 (100%) | 14 (78%) | 38 (78%) |

Abbreviations: BMI, body mass index; CTLA-4, cytotoxic T-lymphocyte–associated antigen 4; ECOG PS, Eastern Cooperative Oncology Group performance status; INF, inflammatory MT; LIV, liver-like MT; MES, mesenchymal-like MT; MT, molecular type; PD-(L)1, programmed death-ligand 1; PRO, proliferative MT.

Table S1B: Significance (p-values) of pairwise comparisons.

| Patient characteristic | MES vs PRO | MES vs INF | MES vs LIV | PRO vs INF | PRO vs LIV | INF vs LIV |
| --- | --- | --- | --- | --- | --- | --- |
| Age, years |  |  |  |  |  |  |
| Continuous | 0.3733 | 0.2844 | 0.8653 | 0.9839 | 0.7849 | 0.8655 |
| Sex |  |  |  |  |  |  |
| Female vs Male | 0.4572 | 1 | 0.6499 | 0.7106 | 1 | 1 |
| ECOG PS |  |  |  |  |  |  |
| 0 vs 1 | 0.4425 | 1 | 0.6169 | 0.4425 | 1 | 0.6169 |
| BMI |  |  |  |  |  |  |
| Continuous | **0.0037**** | **0.1005*** | **0.1673*** | 0.4149 | 0.4228 | 1 |
| Prior lines of therapy |  |  |  |  |  |  |
| Continuous | 0.3660 | 0.6202 | 0.7971 | 0.9656 | 0.6181 | 0.5226 |
| ≥3 vs <3 | 1 | 0.6404 | 0.5088 | **0.1843*** | 1 | 0.2451 |
| Prior anti–*PD-(L)1/CTLA-4* |  |  |  |  |  |  |
| Yes vs No | **0.1297*** | **0.0372**** | **0.1228*** | 0.4181 | 1 | 0.6562 |

Abbreviations: BMI, body mass index; CTLA-4, cytotoxic T-lymphocyte–associated antigen 4; ECOG PS, Eastern Cooperative Oncology Group performance status; ICI, immune checkpoint inhibitor; INF, inflammatory MT; LIV, liver-like MT; MES, mesenchymal-like MT; MT, molecular type; PD-(L)1, programmed death-ligand 1; PRO, proliferative MT.

** p < 0.05

* p < 0.2

Continuous values: Mann-Whitney U-test; Categorical values: Fisher’s exact test

**Comment:** Higher BMI and proportion of patients with prior anti–*PD(L)-1/CTLA-4* prior treatment in MES vs PRO/INF/LIV.

**Supplementary Table S2: Gene lists of the SOM-modules D1-D28.**

- Found in Supplementary File 2 (Excel)

**Supplementary Table S3: Differentially expressed genes in ON-PRE comparison.**

- Found in Supplementary File 3 (Excel)

**Supplementary Table S4: Multivariate Cox proportional hazards model for MTs (PRO+LIV vs. INF+MES, reference is INF+MES), active liver metastases (LM_Y vs. LM_N), and ECOG performance status (0 vs. 1).**

| **Variable adjusted for:** | **Hazard Ratio** | **CI lower** | **CI upper** | **p-value** |
| --- | --- | --- | --- | --- |
| **None (univariate)** | 2.17 | 1.05 | 4.48 | 0.0372 |
| **ECOG** | 2.89 | 1.33 | 6.32 | 0.0077 |
| **Liver metastases** | 1.71 | 0.79 | 3.74 | 0.1752 |
| **ECOG + liver metastases** | 2.29 | 1.00 | 5.23 | 0.0498 |

Abbreviations: ECOG, Eastern Cooperative Oncology Group; INF, inflammatory MT; LIV, liver-like MT; LM, liver metastases; MES, mesenchymal-like MT; MT, molecular type; N, no; PRO, proliferative MT; Y, yes.

**Comment:** MT grouping (PRO+LIV vs. INF+MES) remained associated with OS after adjustment for ECOG. When active LM was included, the MT effect was attenuated, consistent with the close relationship between the LIV transcriptional state and liver metastatic disease/biopsy site (i.e., LIV samples derive exclusively from liver biopsies), which introduces collinearity between these variables. Importantly, in the model including both ECOG and active LM, MT continued to contribute prognostic information beyond these clinical covariates.

**Supplementary Figures S1 – S24**


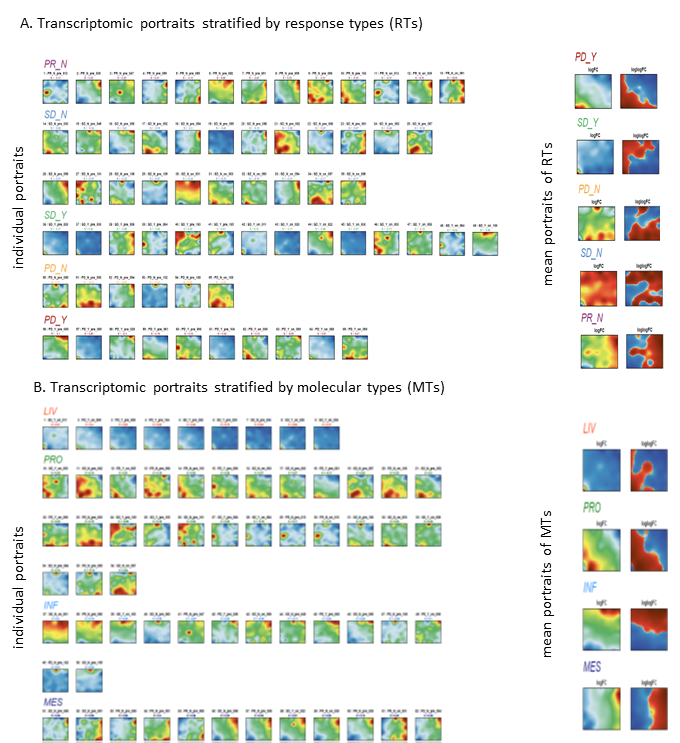


**Figure S1: SOM portrayal of the C-800-01 MSS mCRC cohort.** Each tumor is visualized by its transcriptome portrait representing a 40x40 metagene image. They are stratified according to their response (A) and molecular types (B). Mean portraits for each RT and MT were obtained as averages of the individual portraits in the respective group. Mean portraits were colored in two ways: a) The standard portraits use a red-green-blue scale for maximum-intermediate-minimum expression levels of the genes, respectively. It thus highlights regions of large and low expression. b) The "waterline" portraits color regions of expression higher (red) or lower (blue) than the mean expression of the respective genes averaged over all samples. Note that expression values are normalized and centered, the waterline portraits thus highlight larger areas affected by small expression deviations from their average values. This representation reveals that MTs are characterized by specific regions of gene activation, namely in the left lower corner of the map (LIV), the region below the left-up to right-below diagonal (PRO), the region along the upper edge (INF), and along the right edge (MES). The portraits of RT show correspondence of PD_Y and SD_Y (active liver metastases) with LIV, of SD_N with INF, and of PR_N with MES.


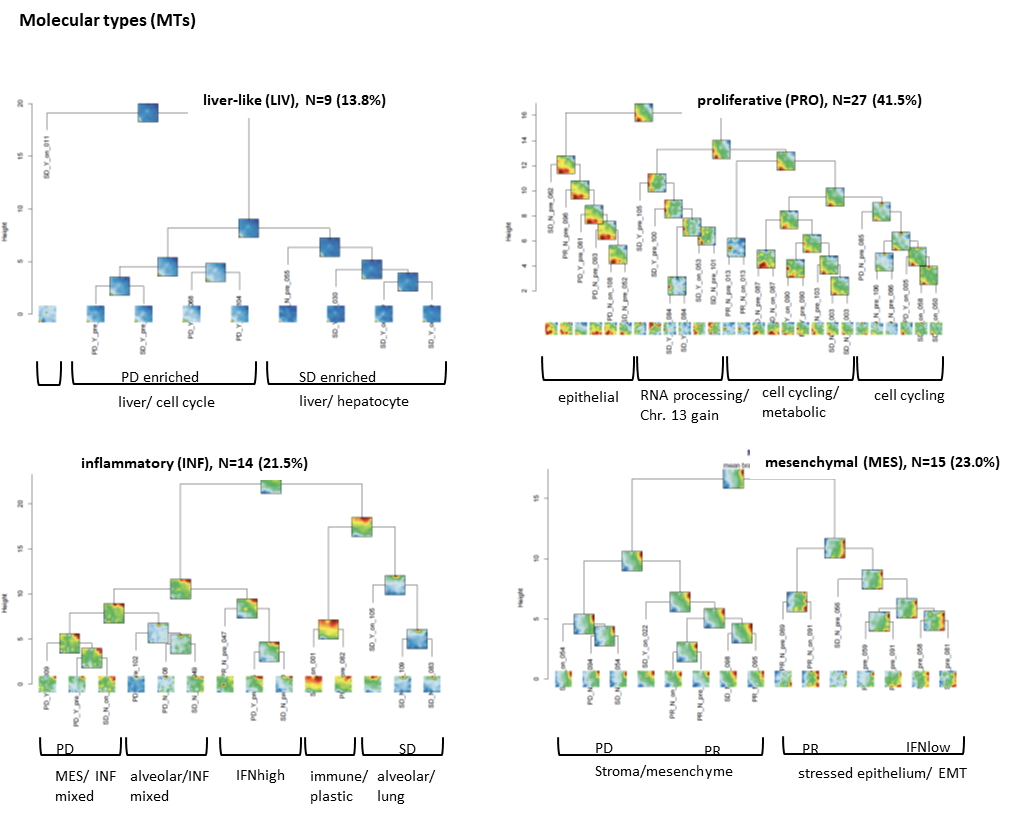


**Figure S2:** **Hierarchical subclustering of the MTs with individual tumor resolution.** Hierarchical clustering of the transcriptomic portraits of the tumors by MT. The functional context annotated below each cluster is based on the oposSOM module annotations.


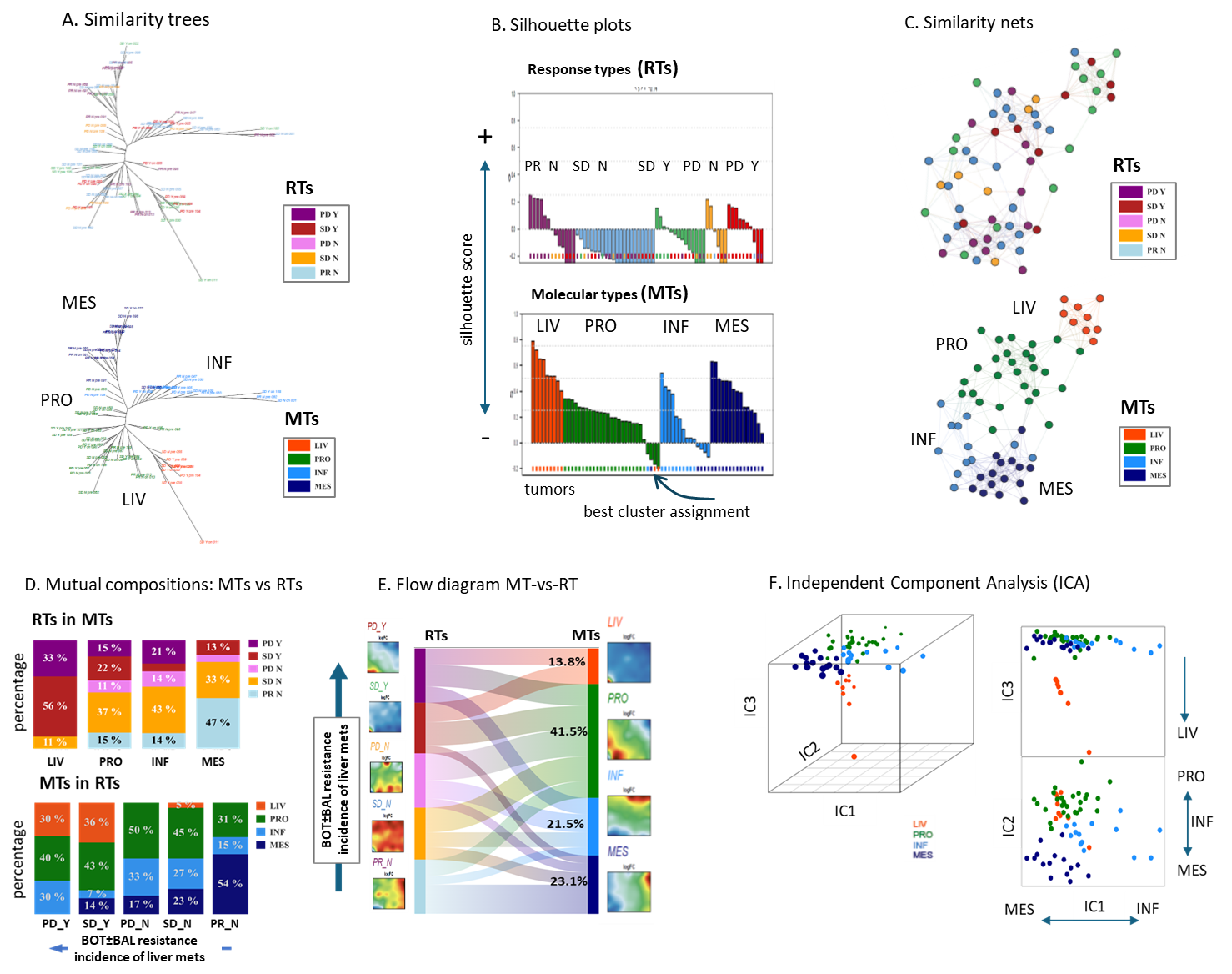


**Figure S3:** **Similarity analysis of the SOM portraits of the tumor samples.** (A) The similarity tree of tumor transcriptomes is displayed, with tumors colored as response types (RTs) in the upper section and as molecular types (MTs) in the lower section. The distribution of tumors along the four major branches of the tree was used for stratifying them into MTs, assigned as Liver-like (LIV), Proliferative (PRO), Inflammatory (INF), and Mesenchymal (MES). (B) The silhouette score estimates the quality of classification [20, 21]. Silhouette plots for the two classification schemes indicate predominantly positive values of the silhouette score for MTs compared with RTs and thus improved clustering resolution. The MTs thus provide a better grouping of the tumors compared with the stratification into RTs. (C) The tumors in the similarity nets were colored according to their RT and MT assignments. MTs show the better clustering. (D) Mutual composition bar plots show a progressive accumulation of tumors with active liver metastases (PD_Y, SD_Y) within the LIV MT, and tumors with partial responses (PR_N) within the MES MT. Hence, the incidence of active liver metastases (LM) and of resistance to BOT±BAL immunotherapy increases from the MES and INF towards the PRO and LIV tumors. (E) A Sankey diagram illustrates the composition flows between RTs and MTs. Comparison of MT and RT reveals accumulation of worse BORR for LIV and PRO tumors compared with INF and MES tumors. Self-organizing map (SOM) portraits of RTs and MTs highlight areas of gene module overexpression within specific regions. (F) Independent component analysis (ICA) plots reveal the separation of MTs along the first three independent components (IC1–IC3), underscoring different molecular characteristics of each subtype. ICA revealed a continuum of molecular states of the PRO, INF, and MES MTs within plane spanned by the independent components IC1 and IC2 while LIV tumors markedly aligned perpendicularly along IC3, suggesting distinct gene expression characteristics. The partly overlapping distributions of the PRO, INF, and MES tumors are due to the heterogeneity of tissue samples and/or continuously changing compositions, e.g., of the TME and/or transition states of the of the tumor cells (see [10, 22, 23] for a detailed discussion).


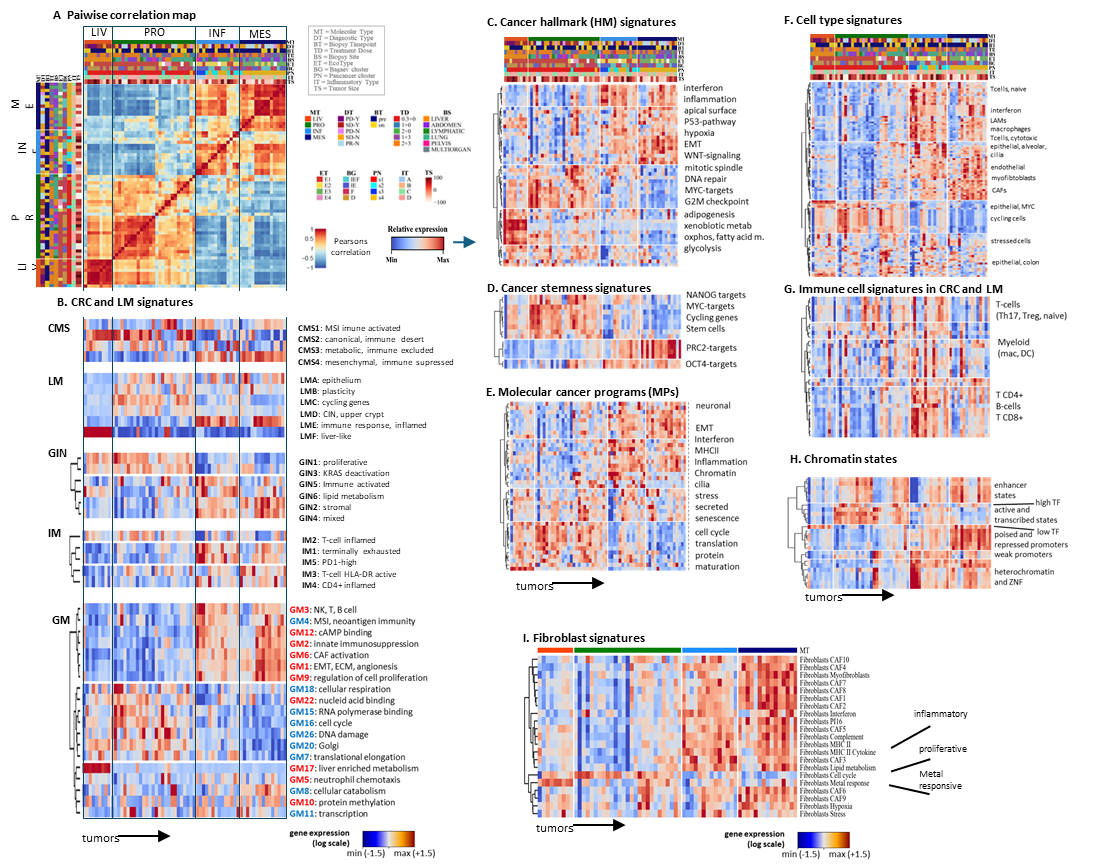


**Figure S4:** **Molecular and functional heterogeneity of the MTs.** (A) The pairwise correlation map identifies two major, mutually anticorrelated clusters including the INF and MES MTs on one hand, and PRO and LIV, on the other one. (B) Gene expression profiles of CRC consensus molecular subtypes (CMS) and CRC liver metastases (LM) taken from [24, 25] demonstrate associations with the MTs. Additional CRC classification schemes such as the gene perturbation interaction network (GIN) [26], immune microenvironment types (IM) [27], and gene modules (GM) of primary CRC and matched LM as suggested in [28] support the MT stratification. GM are marked in red and blue for signatures correlating and anticorrelating with active metastases in the liver, respectively. Analysis of liver metastasis classification signatures [25] revealed that LM_A (mucosal features) and LM_C (cycling gene expression) were associated with the PRO MT, LM_E (inflammatory phenotype) with the INF MT and to a lesser extent with MES, and LM_F (liver-like features) with the LIV MT. Similarly, gene interaction network (GIN) classifications for CRC [26] mirrored these associations, as did immune microenvironment (IM) types [27], particularly those aligning with the INF and MES MTs. These findings suggest that previously described CRC and LM molecular signatures are preserved across the various metastatic biopsy sites in this study. (C)-(H) Heatmaps of the expression signatures of different categories assign functional themes and reveal a large heterogeneity of molecular states: (C) Cancer hallmarks [29], (D) cancer stemness-signatures [30], (E) molecular cancer programs [31], (F) cell-type signatures taken from pan-cancer single cell data [31], and (G) immune cell signatures taken from single cell data of mCRC and LM [32]. (H) Chromatin states of healthy colon [33] and transcription factor (TF) activity categories related to active (high expression TF) and repressed (low expression TF) [34]. (I) Fibroblast signatures extracted from single cell pan-cancer cancer data [31] upregulate in the MES, and to a lesser degree in the INF MTs except that of proliferating and metal-responding fibroblasts.


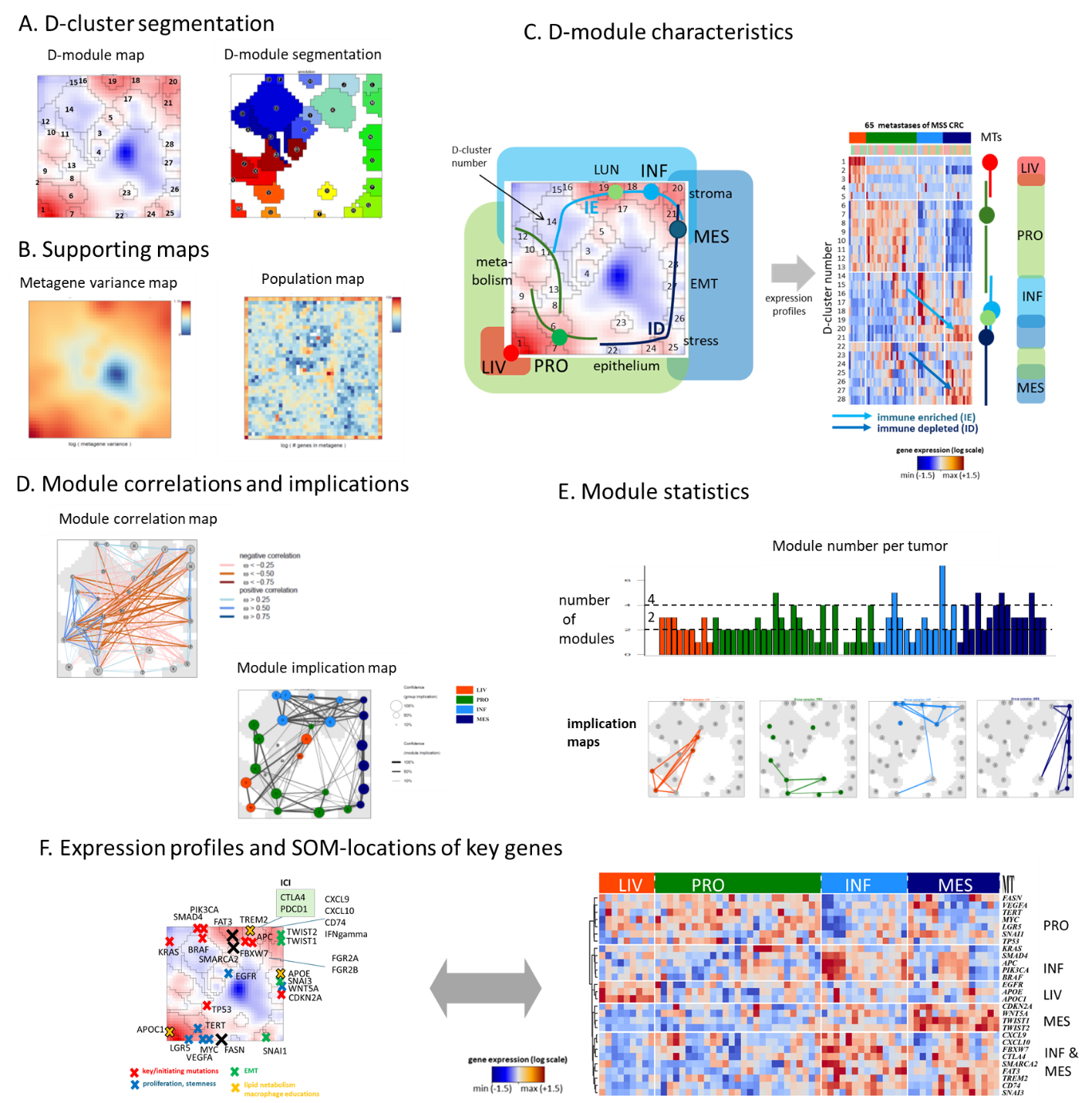


**Figure S5:** **Topology of the transcriptional SOM landscape of metastases arising from MSS mCRC.** (A) We employ a distance (D) map visualization, which identifies gene modules as clusters based on the variation in distances between their expression vectors across the map. The D-cluster map visualizes the mutual distances between adjacent metagene pixels in Euclidean distance space (blue-white-red refers to large-intermediate and small distances) where clusters were identified by the white "halo" as shown in the segmentation map. (B) The metagene variance map shows that genes with most variable expression (brown) locate in the left lower and right upper part of the SOM referring to overexpression in the LIV/PRO and INF/MES MTs, respectively. Virtually invariant genes accumulate in the blue area. The population map color-codes number of gene transcripts per metagene/pixel. (C) Overall, we identified 28 D-clusters that represent modules of coregulated genes numbered from D1 to D28. They can be arranged along "trajectories" of similar expression profiles which assign to the four molecular types (see also the heatmap in the right part). Note that the INF (light blue) and MES (dark blue) trajectories reflect immune-enriched (IE, hot) and -depleted (ID, cold) transcriptional states linking PRO and INF/MES types (see arrows in the heatmap). (D) The spot correlation map visualizes correlations between the expression profiles of the spot modules. Spot-modules upregulated in PRO anticorrelate with modules upregulated in INF and MES (red lines). Modules upregulated in LIV form a separate entity. The implication map connects spot-modules jointly expressed in the individual portraits. Jointly expressed spots agree roughly with the trajectories defined in part C. (E) The number of spots per individual portrait varies between 1 and 6 where MES tumors show the largest number of 3-4. The maps below show spot implications for each MT. Notably spot 23 combines with other spots in the PRO, INF, and MES MTs (see also the profile of spot 23 in the heatmap in part (C), which suggests its independent origin presumably related to colon epithelium. (F) SOM locations (left part) and expression heatmap of a series of CRC key genes. Initiating mutations (red crosses) locate the upper edge (except TP53) and upregulate in INF-tumors while TP53 upregulates in PRO-tumors together with MYC, LGR5, and TERT.

**Summary:** The self-organizing properties of SOM machine learning generate a topology-aware transcriptomic landscape of MSS mCRC tumors, which integrates molecular functions, TME-ecosystems, biases due to biopsy sites as well as the contribution of these variables. The landscape divides into four major regions referring to an increased content of proliferating tumor cells, to immuno-suppressive LM, to mesenchymal and immuno-depleted fibrotic areas and to immunogenic metastases.


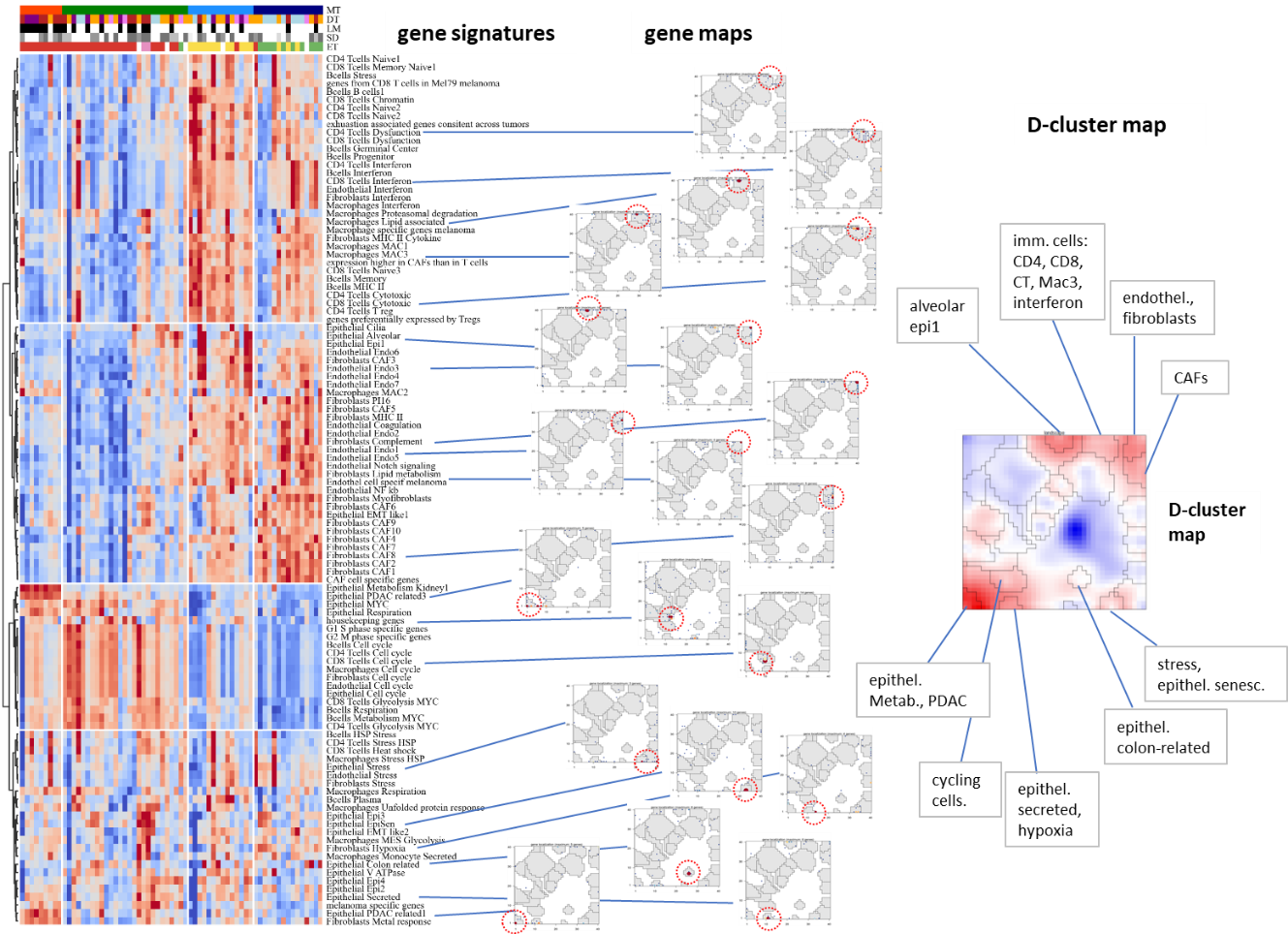


**
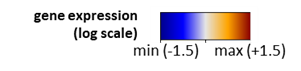
**

**Figure S6: Cell type related expression signatures translate into characteristic patterns of the gene maps.** The signatures, taken from [31], characterize the transcriptional intratumor heterogeneity extracted from a large pan-cancer study. The gene maps show the distribution of selected signatures in the SOM. The red circles indicate areas of accumulation of the respective genes (dots). The grey areas refer to D-clusters. The D-cluster map and a summary of functional regions is shown in the right part.


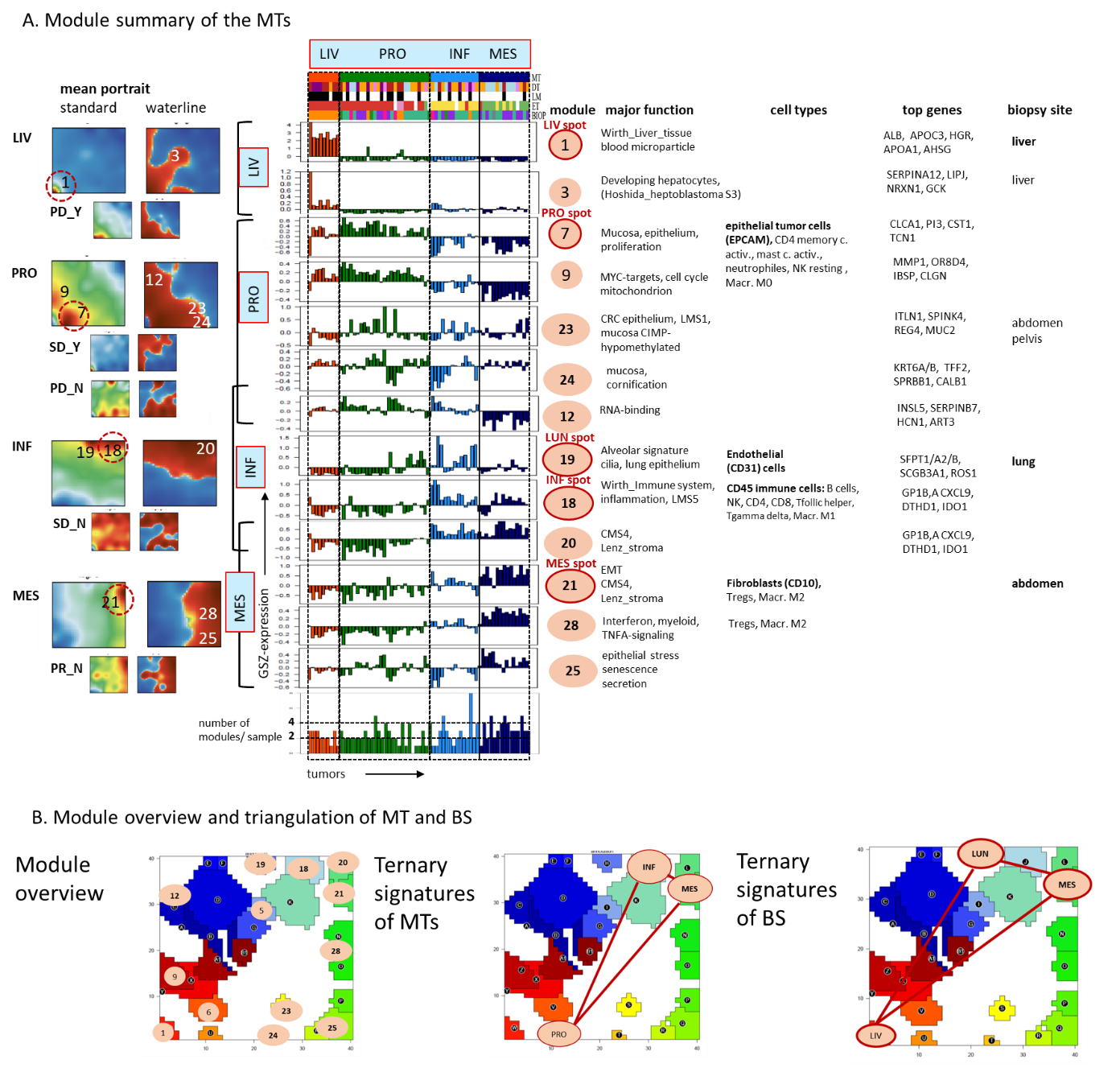


**Figure S7:** **Module of coregulated genes extracted from the SOM portraits.** (A) Mean portraits (left part, see also **Figure S1**) were segmented into (red) spot like areas referring to modules of correlated genes upregulated in the respective MT (or RT, small portraits). Modules were obtained using D-segmentation [8] and labeled with numbers D1-D28 (not all modules were considered here). Expression profiles are shown by bar plots. Major functional context, related cell types, key genes, and associated biopsy sites are shown in tabular form. Major functions were obtained using gene set overexpression analysis of the module-genes as implemented in oposSOM [4, 6, 8]. (B) The overview map shows the modules considered. Three modules span the ternary coordinate system of the MTs PRO-INF-MES and another three modules the ternary coordinate system of the biopsy sites LIV-LUN-ABD(=MES). The former one characterizes the tumor-intrinsic properties while the latter one characterizes the specific properties of the biopsy site. Note the identity of the LIV-tissue and LIV-MT modules and of the ABD-tissue and MES-MT modules while the LUN-tissue and PRO-MT modules are different.


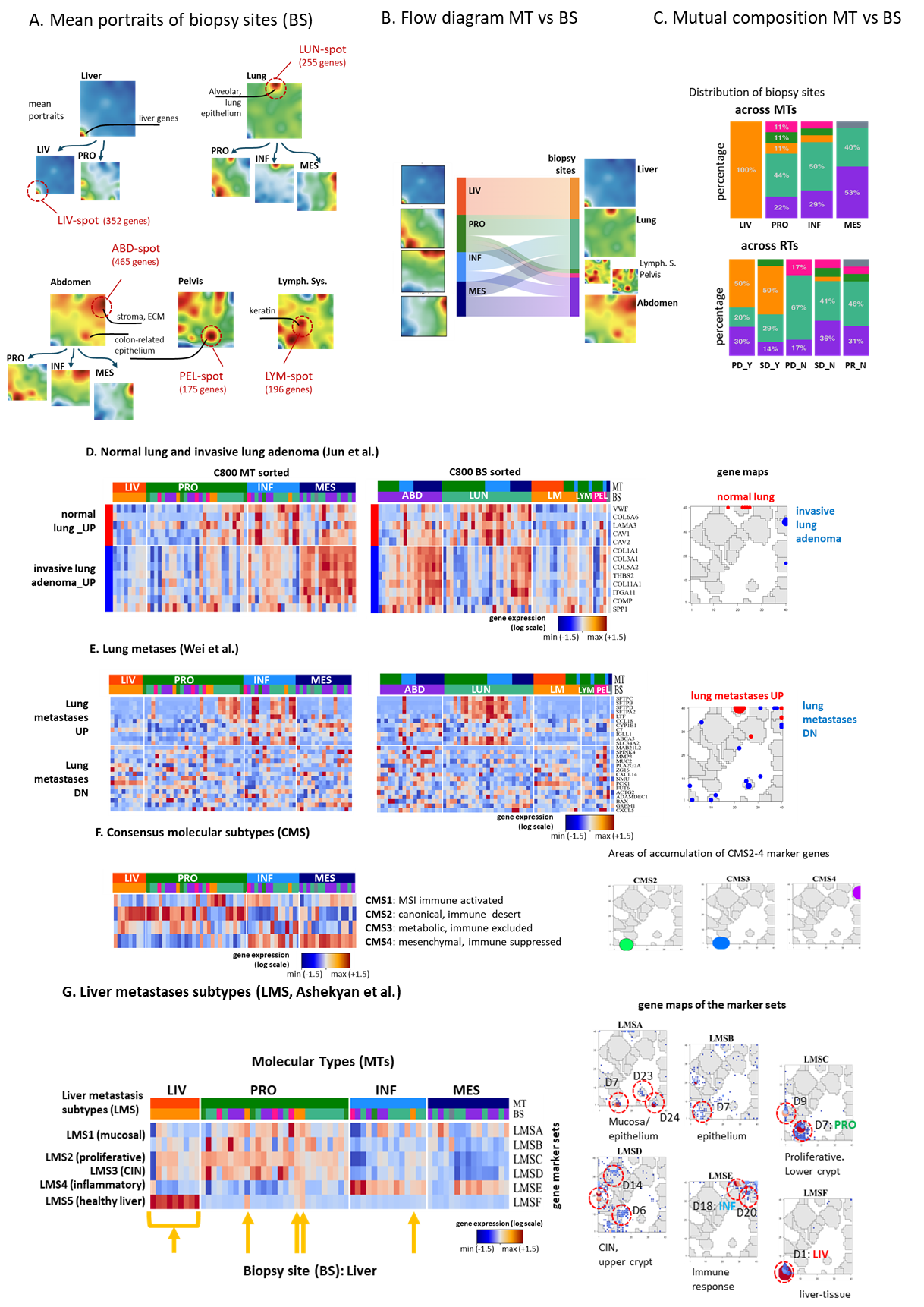


**Figure S8:** **Transcriptomic characteristics of biopsy sites of CRC metastases.** (A) **Mean portraits of the biopsy sites** indicate tissue specific transcriptional programs and common programs shared between the biopsy sites especially in the MT-stratified portraits. The biopsy site–stratified mean portraits reveal specifically upregulated "tissue"-spots which can be assigned to liver functions for LM [25] (LIV-spot), alveolar, normal lung tissue [35], and lung metastases of CRC [36] (LUN spot), and endothelial, stromal, CMS4-like characteristics of abdominal metastases [37] (ABD spot). These biopsy site specifics of the transcriptomes can be partly explained by normal tissue contaminations from the biopsy sites because tumor samples were extracted without microdissection. Also, specific adaptations of tumor cells to their environment should be considered, e.g., of the tumor-specific expression of keratin genes such as KRT-20/19/7 in lymph node metastases [38, 39] associating with cystic degeneration, of lung surfactant genes such as SFTP-C/B/A2/D genes in lung metastases [36], and the specific mesenchymal, CMS4-like transcriptomic characteristics related to epithelial adaptation of abdominal metastases in the peritoneum which were partly perceived as distinct disease entities [37, 40, 41]. Stratification of biopsy portraits with respect to PRO, INF, and MES MTs shows the underlying MT-specific spots as cross–biopsy site tumor characteristics. (B) **Flow analysis of the biopsy site indicated** slight organotropism of INF tumors towards LUN biopsy site and of MES towards ABD. (C) **Distribution of biopsy sites across the MTs and vice versa.** We evaluated how biopsies from different primary and metastatic sites (biopsy sites) distributed across the RT and MT. As expected, biopsies from the liver dominate in the LIV reflecting the fact that the liver-like characteristics of this MT are partly due to contaminations of tumor samples with adjacent liver tissue [25]. Metastases from the other biopsy sites distribute across the RTs and MTs with a slight accumulation of abdominal biopsy site in the MES MT while lung metastases slightly accumulate in the INF MT. (D) **Gene markers of normal lung tissue and or invasive lung adenoma** taken from [35] upregulate in LUN-metastases of CRC of the INF and PRO MTs and in MES MT where the MES-related markers also upregulate in ABD-metastases of the MES MT. (E) **Differential marker genes of lung metastases-vs-pCRC** taken from [36] show similar expression patterns as healthy lung tissue regarding, e.g., genes encoding lung surfactants such as SFTP-B/C/D/A2 related to surfactant metabolism. Both healthy lung specific and lung metastasis genes accumulate in the LUN-spot while genes of invasive lung adenoma are found in the MES/ABD spot upregulated in ABD metastases of this study. (F) **Markers of consensus molecular subtypes of CRC CMS1-CMS4** [25] accumulate in the regions of the PRO (CMS2 and CMS3) and MES/ABD (CMS4) spots. Abdominal/peritoneal metastases of CRC represent a distinct CMS4, epithelial intrinsic subtype expressing the marker gene MSH [37] located in D21 module referring to a fibrotic-inflammatory immunotype. (G) **Gene expression signatures of liver metastasis subtypes (LMS)** were identified in transcriptomes of laser-captured LMs of CRC taken from [25] and mapped into the C-800-01 data. Overall, six signatures (LM A-F) characterize five LMS (LMS1-5). LMS5 refers to healthy liver tissue and overexpresses in all biopsies of LM (yellow arrow below the heatmap) which accumulate in the LIV-MT (composed exclusively of LM biopsies) but occur also in the PRO and INF MTs. All LM biopsies except one) show also moderate activation of signatures which originate from tumor tissue reflecting mucosal (LMS1) and proliferative (LMS2 and LMS3) characteristics. One LM shows inflammatory characteristics which is classified as INF MT. Note that about 24% of a cohort of 283 LM was assigned as inflammatory LMS5 [25]. The maps of the gene signatures on the right reveal accumulation of the genes in different D-modules identified in the C-800-01 cohort thus reflecting mutual agreeing functional context (**Figure S7**).

**Summary:** The transcriptomes of different biopsy sites indicate biopsy site–specific expression modules which originate from contaminations of healthy tissue surrounding the tumors and tumor tissue which shows partly biopsy site–specifics (lung metastases) combined with MS-intrinsic properties (LM and ABD). SOM segmentation in combination with gene signatures separates the respective components on a qualitative level.


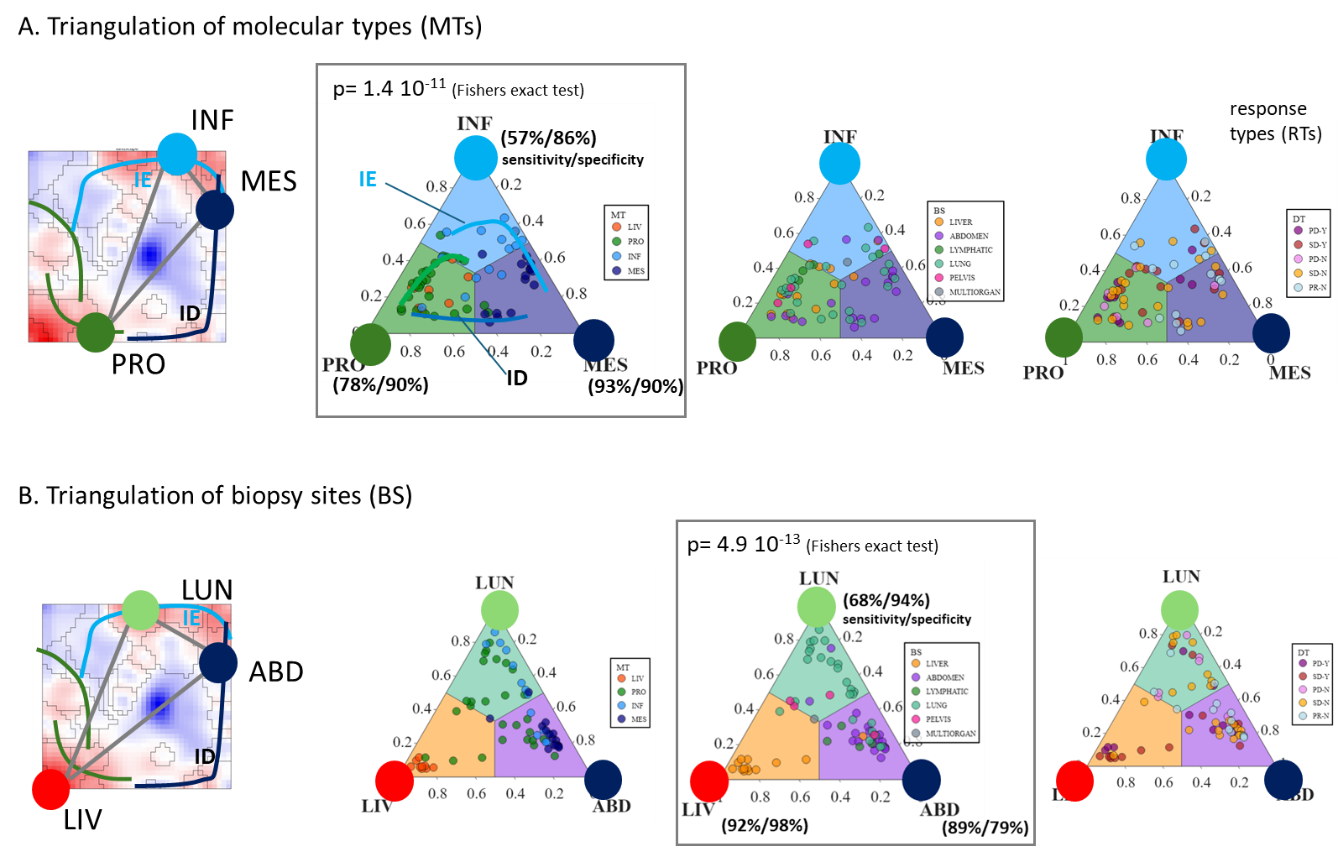


**Figure S9:** **Triangulation of molecular types and biopsy sites.** (A) For triangulation, three core-MT spots labelled as PRO, INF, and MES were selected as indicated in the overview SOM on the left. Gene set expression scores from these spots were used to construct ternary diagrams that effectively segregate the MTs. For comparison, tumor stratifications biopsy site and RT (response type) are shown from left to right. Tumor distributions in the MT diagram align along trajectories corresponding to immune-depleted (ID) and immune-enriched (IE) states, following the PRO-MES and PRO-INF/INF-MES axes, respectively. MT-based triangulation clearly distinguishes tumors along the ID and IE trajectories. (B) Triangulation using core spots corresponding to biopsy sites allows segregation of tumors by metastatic location—specifically, liver (LIV), lung (LUN), and abdominal (ABD) metastases. Overall, triangulation based on gene expression scores of core SOM spots provides a multidimensional coordinate system for comprehensive tumor typing in terms of molecular subclass, TME state, and anatomical biopsy origin. Fisher’s exact test estimated significance of sorting PRO-INF-MES MT tumors in the resp. corners (in A) and of sorting LIV-LUN-ABD biopsy sites into the resp. corners (in B).


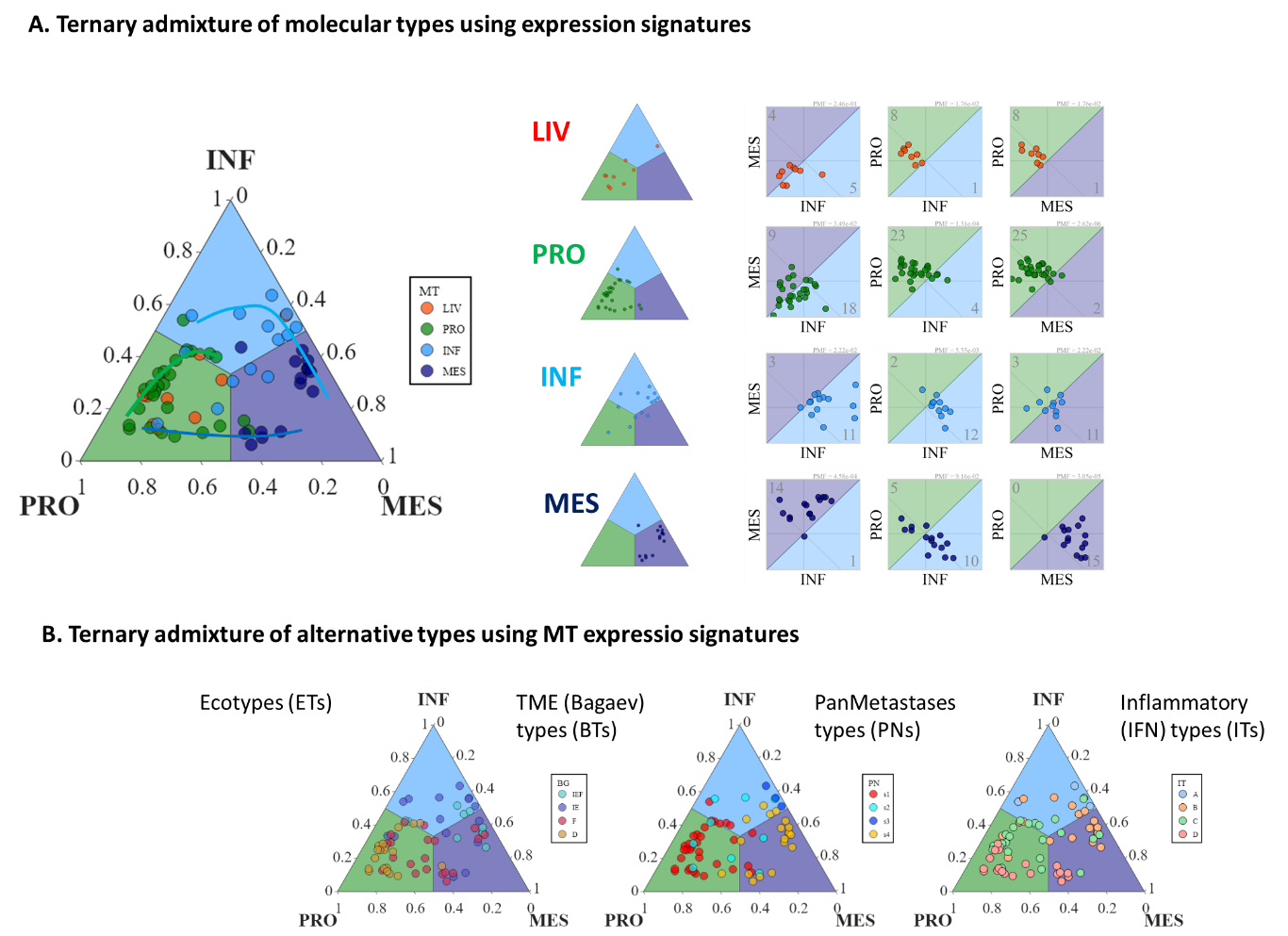


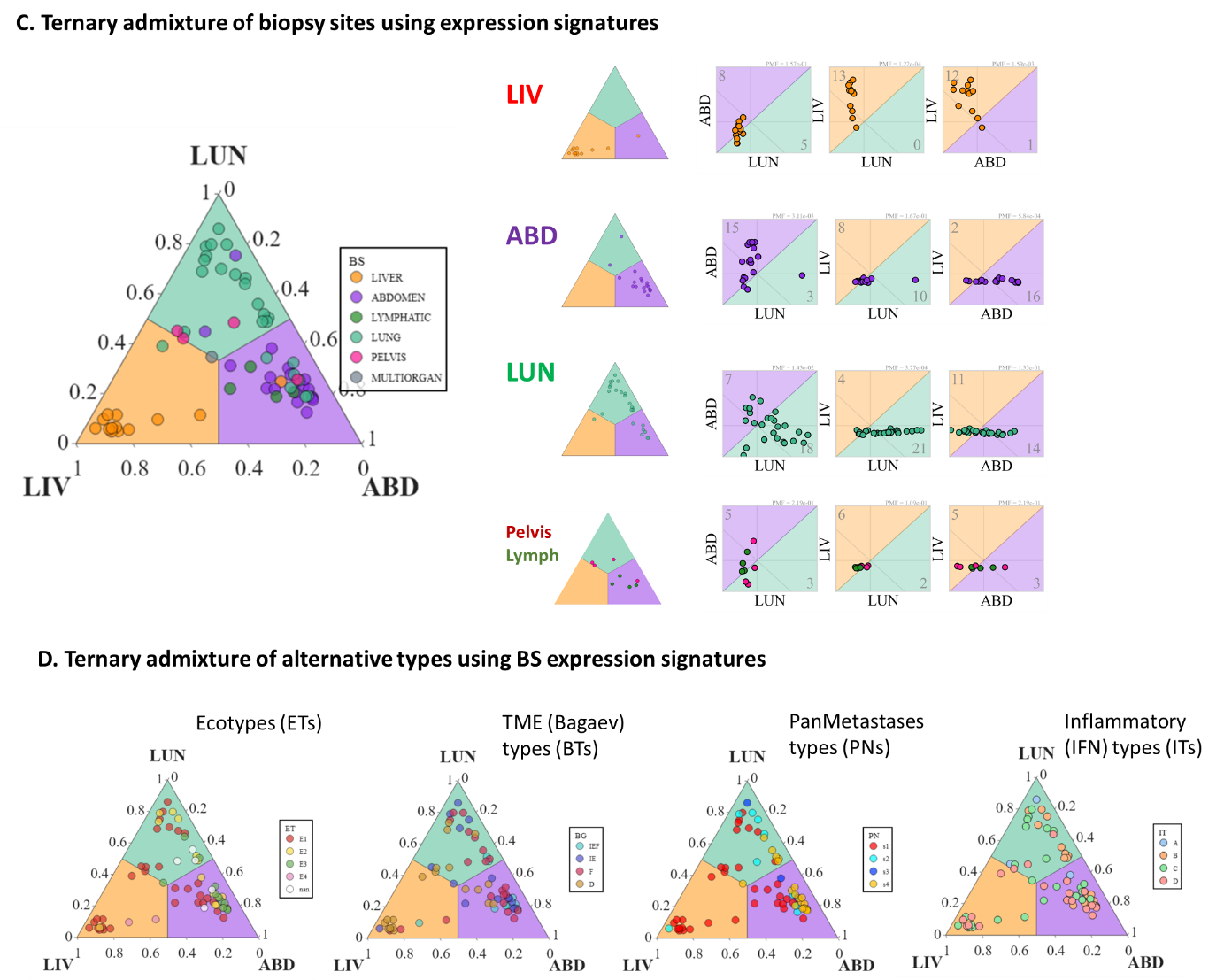


**Figure S10:** **Ternary analysis of MT and biopsy site using expression signatures.** (A) Ternary diagram using expression signatures of the PRO-, INF-, and MES-spots separates tumors into the MTs. The right part separately shows ternary diagrams of tumors of LIV, PRO, INF, and MES MTs as well as biplots of all combinations of scores which reveal association of LIV and PRO tumors with high PRO-score values (criterion: PRO-score is greater than the other scores, of INF tumors with high INF-score values and of MES-tumors with high MES-score values. (B) Ternary diagrams of the alternative tumor types accumulate proliferative and immune depleted subtypes in the PRO-corner, of interferon-high ITs in the INF-corner and of fibrotic BT in the MES-corner thus supporting the triangularization based on the expression scores. (C) Ternary diagrams using the expression signatures of the LUN-, LIV-, and ABD-spots separate the respective metastases. Lung metastases distribute along the LUN-ABD axis in agreement with the transcriptional properties of lung adenoma and lung metastases of CRC of independent studies (**Figure S8A-B**) which show either LUN- or MES/ABD-characteristics where the latter one dominates in invasive adenomas. The LM reveal a distinct transcriptome compared with the other biopsy sites whose transcriptomes do not mix with that of the liver as evidenced by the horizontally grouped tumors in the biplots. The rarer biopsy sites accumulate in the LUN (pelvis) and MES (lymph node) parts of the ternary diagram. (D) Stratification of tumors using the alternative typings mostly does not show a clear separation between the biopsy sites, meaning that all these typings cover different biopsy sites.


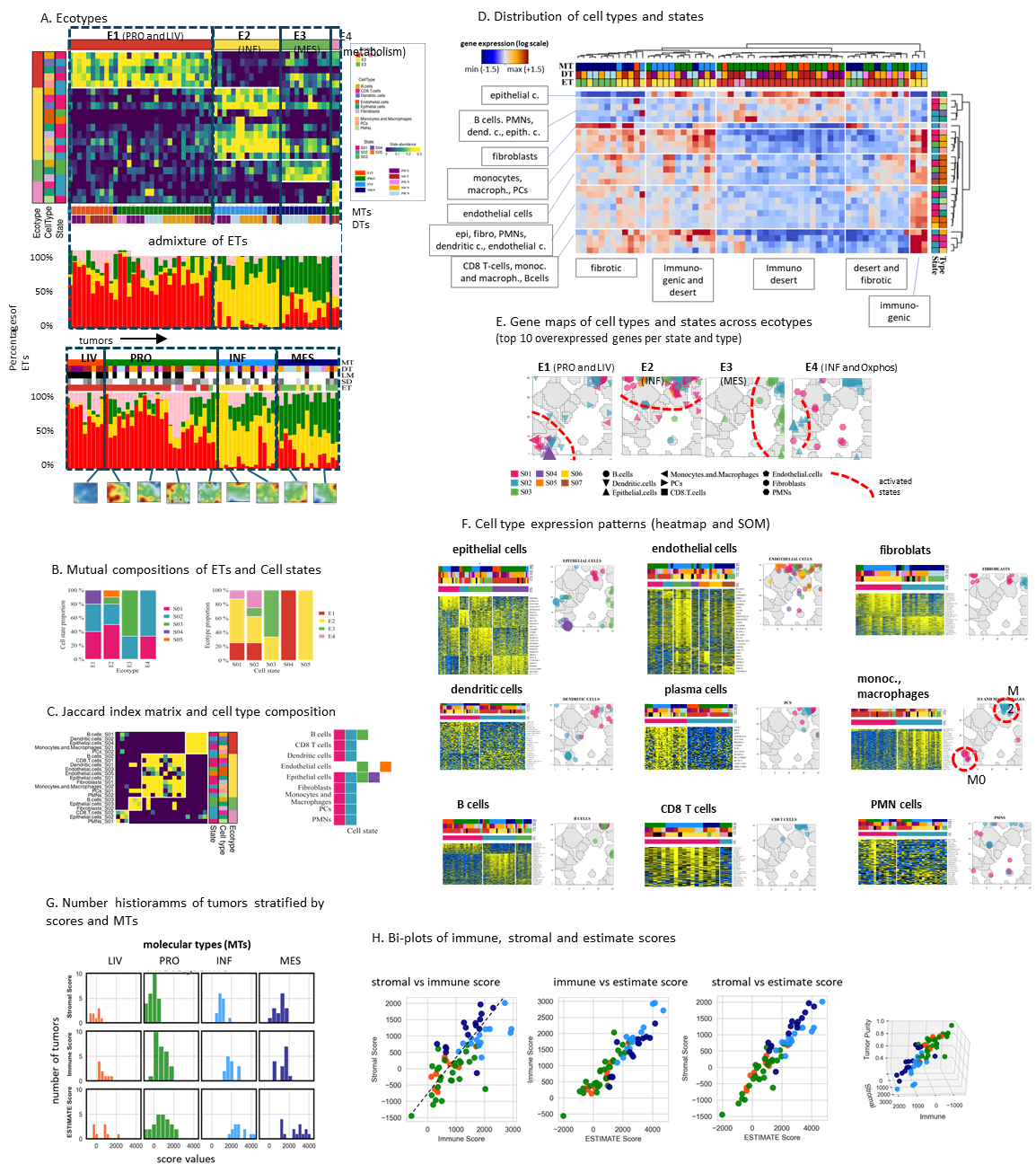


**Figure S11:** **Ecotyping and ESTIMATE analysis of the tumors** using the Ecotyper [17] and ESTIMATE frameworks [18].

**Ecotyping:** (A) Four ecotype-clusters were identified which strongly overlap with the MTs, namely E1 with LIV and PRO, E2 with INF, and E3 with MES, while E4 refers to energy metabolism and includes two PRO tumors. The admixture plot shows that the tumors are composite mixtures of the ET-components where E1 (red) dominates in LIV and PRO, E2 in INF, and E3 in MES. The E4 component significantly contributes to LIV and PRO tumors. It can be associated with energy metabolic functions (oxidative phosphorylation, glycolysis). (B) The ecotypes decompose into cell states (left plot) and vice versa, the cell states distribute across the ETs (right plot). (C) The Jaccard index matrix reveals the cell type composition of the ETs (left plot). The right plot illustrates their distribution across the ETs. (D) The heatmap shows the distribution of cell types and states across the tumors. Clustering decomposes them into TME groups of highly immunogenic, fibrotic, and (immuno-) desert groups and mixtures of them. (E) Gene SOM maps of the top ten upregulated genes of the cell types detected in each ET. The genes accumulate in specific regions corresponding to the MT portraits as indicated by the dashed red lines. Note that different states of each cell type can appear in different regions due to different cell functions. (F) Gene expression heatmaps of different cell types stratified by cell states (blue to yellow color scale for low to high gene expression, respectively) and their maps into the SOM.

**ESTIMATE:** (G) The ESTIMATE analysis predicts sample tumor content (ESTIMATE score, proportionally inversed to tumor cell content), stromal cell content (stromal score), and immune cell content (immune score) [18]. ESTIMATE stromal scores were highest for MES tumors, immune scores for INF tumors, and highest tumors cell content was observed in PRO and LIV tumors. (H) The biplots indicate partly overlapping distributions of INF (light blue) and MES (dark blue) tumors where the former one is shifted toward higher immune scores and the latter one towards higher stromal score values. LIV and PRO tumors are characterized by low immune and stromal score values and high tumor cell content.

**Summary:** EcoTyper and ESTIMATE provided consistent estimations of sample tumor content and TME composition: PRO and LIV samples were characterized by high tumor content while INF and MES showed high immune cells abundance in INF and high stromal cells/fibroblasts in MES.


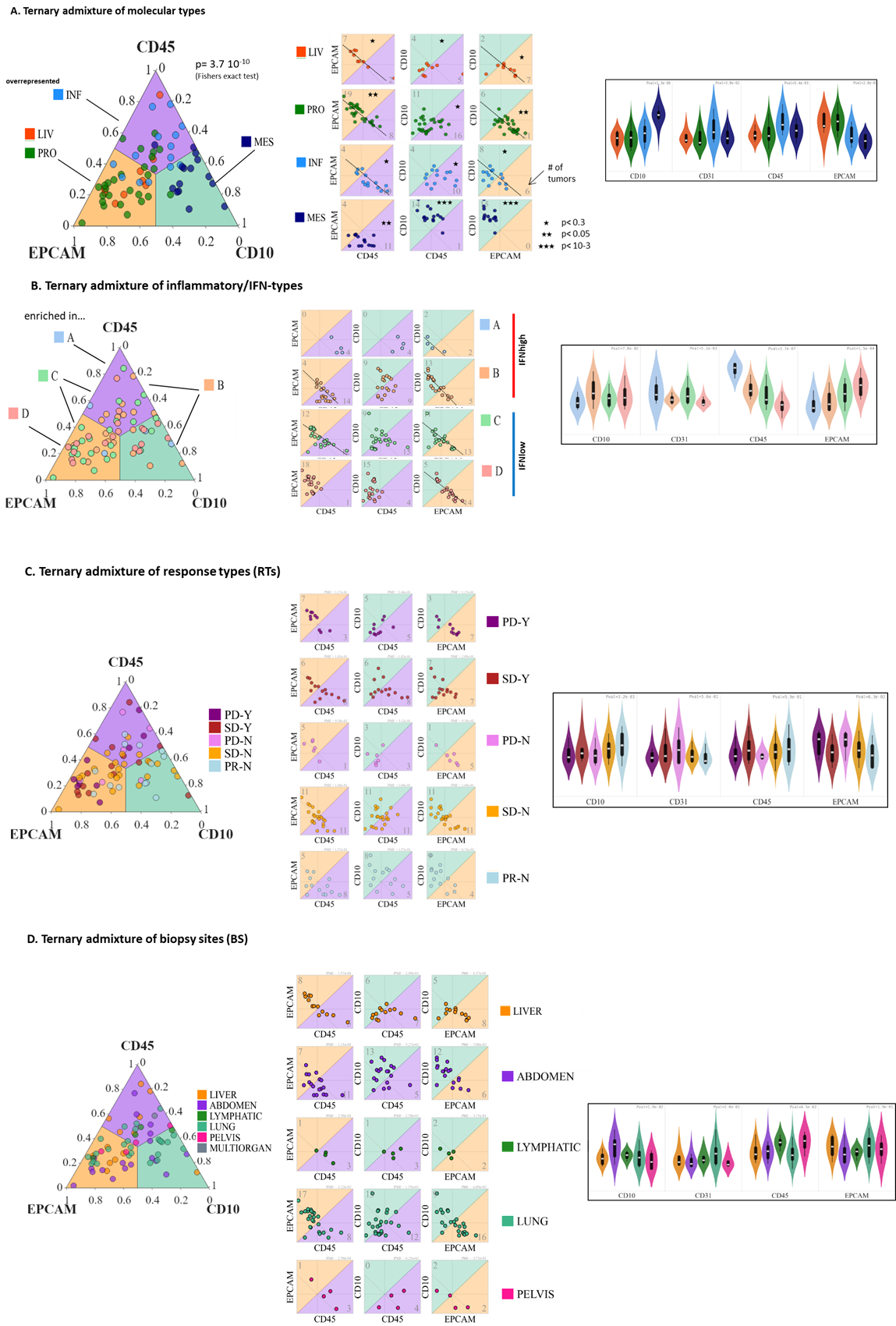


**Figure S12:** **Ternary analysis of cell-type composition analysis** **of EPCAM (epithelial tumor cells), CD45 (immune cells), and CD10 (fibroblasts)** using ternary diagrams, pairwise bi-plots, and violin plots (from the left to the right). The violin plots also show CD31 (endothelial) cells. (A) The ternary composition diagram of the tumors stratified by MTs indicates specific enrichment of CD45 (immune cells), EPCAM (epithelial tumors cells), and CD10 (fibroblast) in different MTs. The right part shows biplots of all combinations of the TME composition. Enrichments in the compartments are indicated (*p < 10^−1^, binomial statistics). (B) Distribution of samples stratified by ITs: High-IFN-type A samples accumulate in the CD45-corner of the ternary diagram, low-IFN type D samples in the EPCAM corner, and intermediate IFN-type B and C along the CD45-CD10 edge and the EPCAM-CD45 edge, respectively, thus reflecting associations between cell compositions and immunogenicity of the tumors. The biplots further confirm these patterns more in detail. EPCAM-vs-CD45 and EPCAM-vs-CD10 reveal negative slopes (see lines), meaning that the amount of tumor cells anticorrelates with immune cells and fibroblasts. The violin plots indicate correlation between CD45 and IFN-score and anticorrelation between EPCAM and IFN-score. (C) Distribution of samples stratified by RTs: RTs more randomly distribute in the ternary diagram. The violin plots indicate decreasing tumor cell content (EPCAM) with improved diagnosis along the PD_N – SD_N – PR_N and PD_Y – SD_Y axes. (D) Distribution of samples stratified by biopsy site: Abdominal metastases show an accumulation of CD10 cells and lung (and to a lesser degree liver) metastases slightly accumulate EPCAM tumor cells while pelvis show and increased fraction of CD45 immune cells.


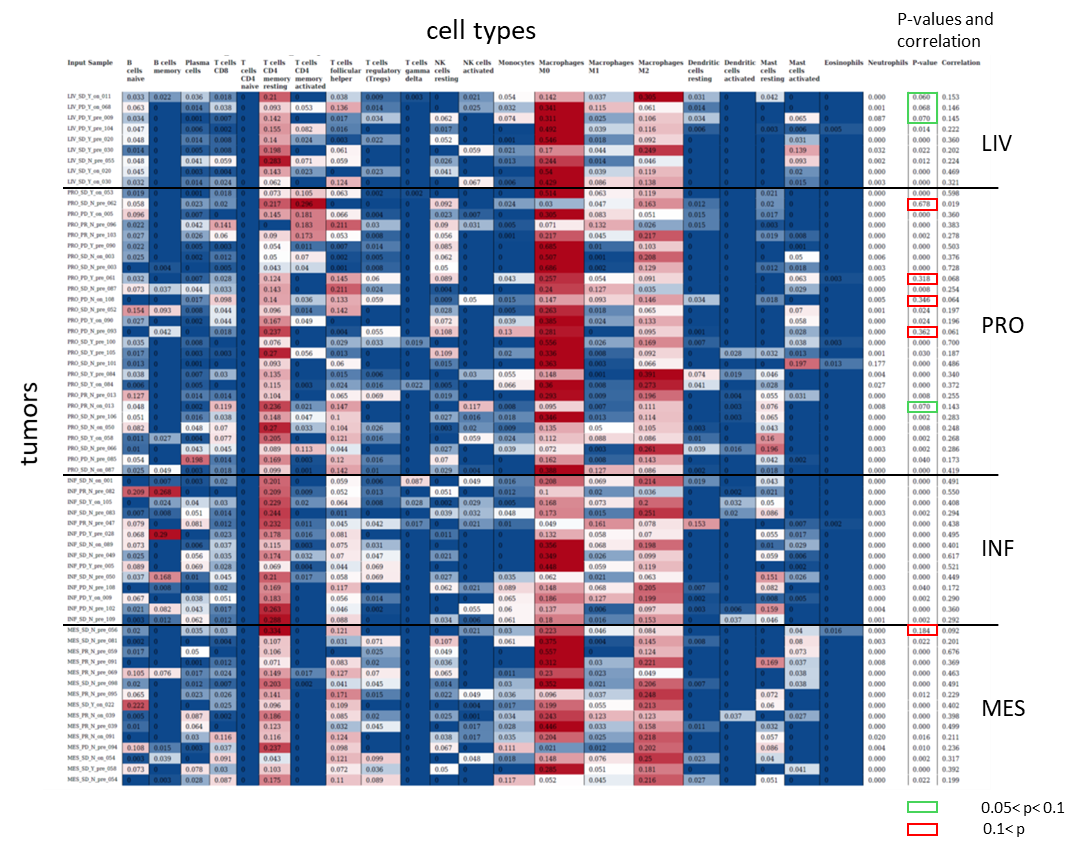


**Figure S13:** **CIBERSORTx LM22 output table [13].** All results are reported as relative fractions normalized to 1 across all cell subsets. The correlation coefficients and p-values estimate the goodness of fit of original and estimated values.


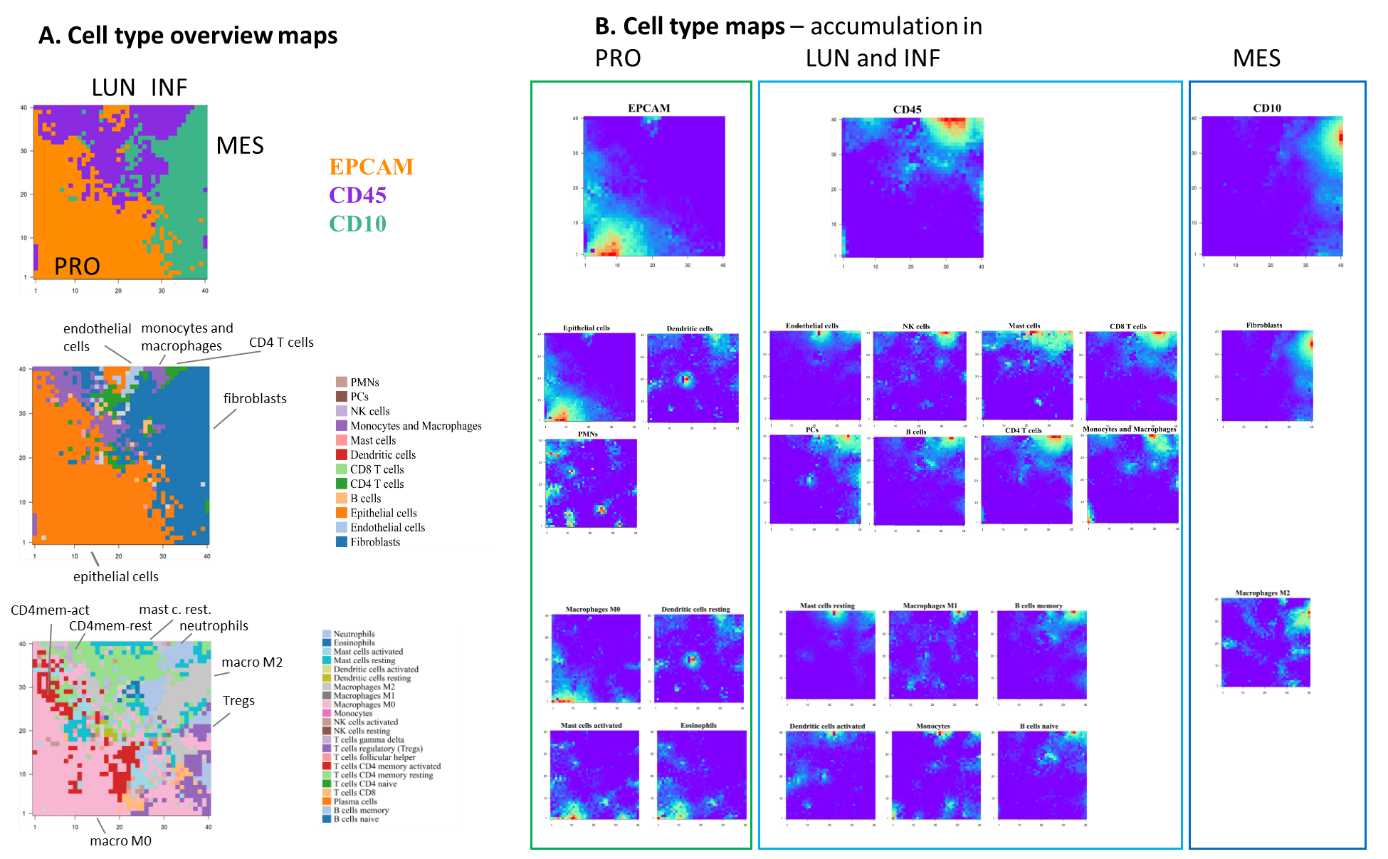


**Figure S14:** **Cell type maps** were obtained by calculating the dot products between the expression profiles of the 40x40 metagenes in the SOM and the profiles of the cell type fractions obtained by CIBERSORTx and subsequent color-coding the metagenes by the resulting values. Pixels with high dot products were colored in red-to-light blue indicating high association between the expression and cell type fraction. Each cell-type is characterized by a localized area of high correlation with metagenes in the map. (A) The overview maps represent the cell type of maximum correlation in each metagene. (B) Individual cell type maps sorted by accumulation in the PRO-, INF-, and MES-module regions. High correlation regions were found in the area of the INF-module for a series of CD45 immune cells such as CD4+ and CD8+ T cells, follicular helper cells, gamma delta cells, NK cells, activated dendritic cells, and pro-inflammatory/antitumoral M1 macrophages [42]. CD10 fibroblasts, regulatory T cells and anti-inflammatory/protumoral M2 macrophages correlate with the MES-module region upregulated in MES tumors. EPCAM tumor cells, CD4 memory T cells, activated mast cells, and naïve (M0) macrophages were found to correlate with the PRO-module expression. Hence, SOM portrayal thus integrates cell type information with underlying molecular functions in the SOM transcriptome landscape.


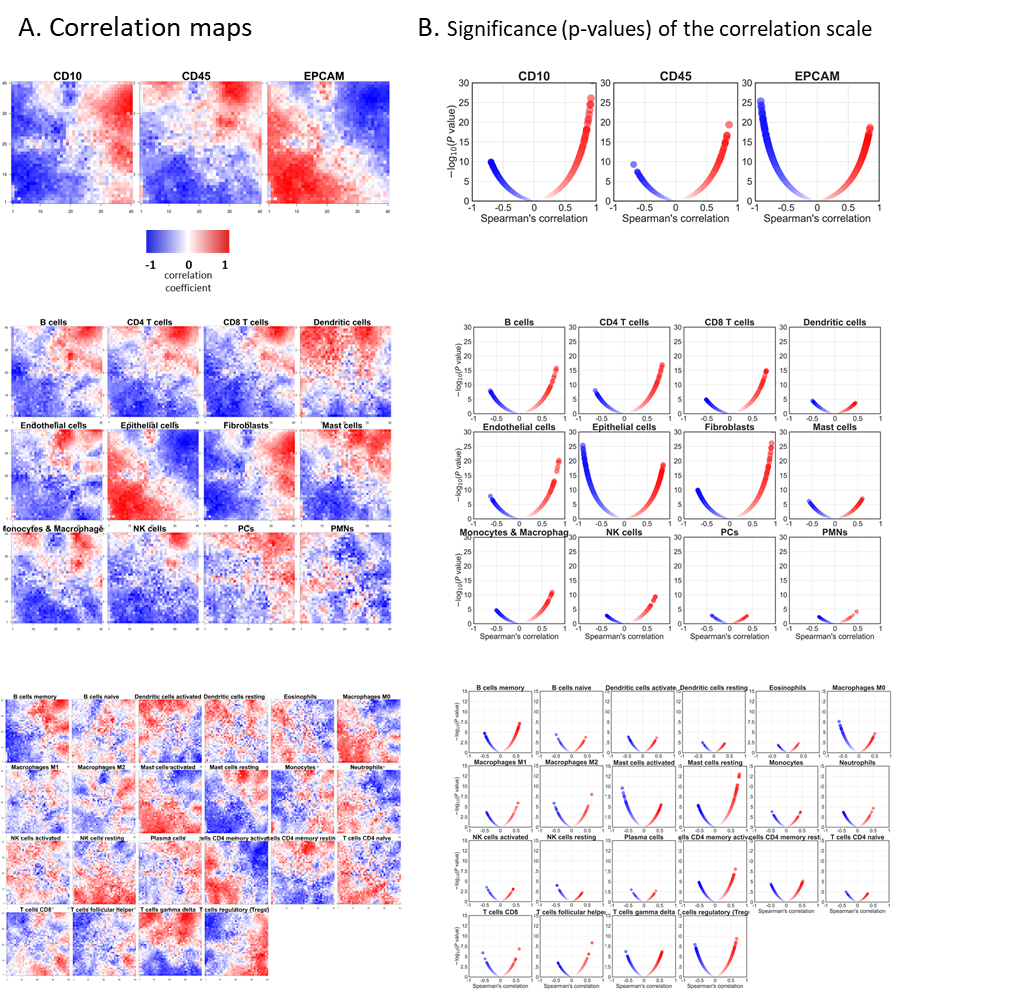


**Figure S15:** **Correlation maps of cell types and the significance of the correlation scale.** (A) Correlation maps of the cell types were obtained by calculating the Spearman’s correlation coefficient between the expression profile and the cell type profile in each metagene. They directly relate to the dot-product maps and are grouped as in **Figure S14**. (B) The significance scale for each cell type is shown as the p-values plotted as a function of the correlation coefficient for each cell type. Minimum p-values refer to the metagenes of highest absolute values of the correlation coefficient. For major cell classes (EPCAM, CD10, and CD45), we found highly significant associations between gene expression and cell type fractions (p < 10^−20^) and for immune cells (LM22), a typical range of 10^−10^< p < 10^−3^.


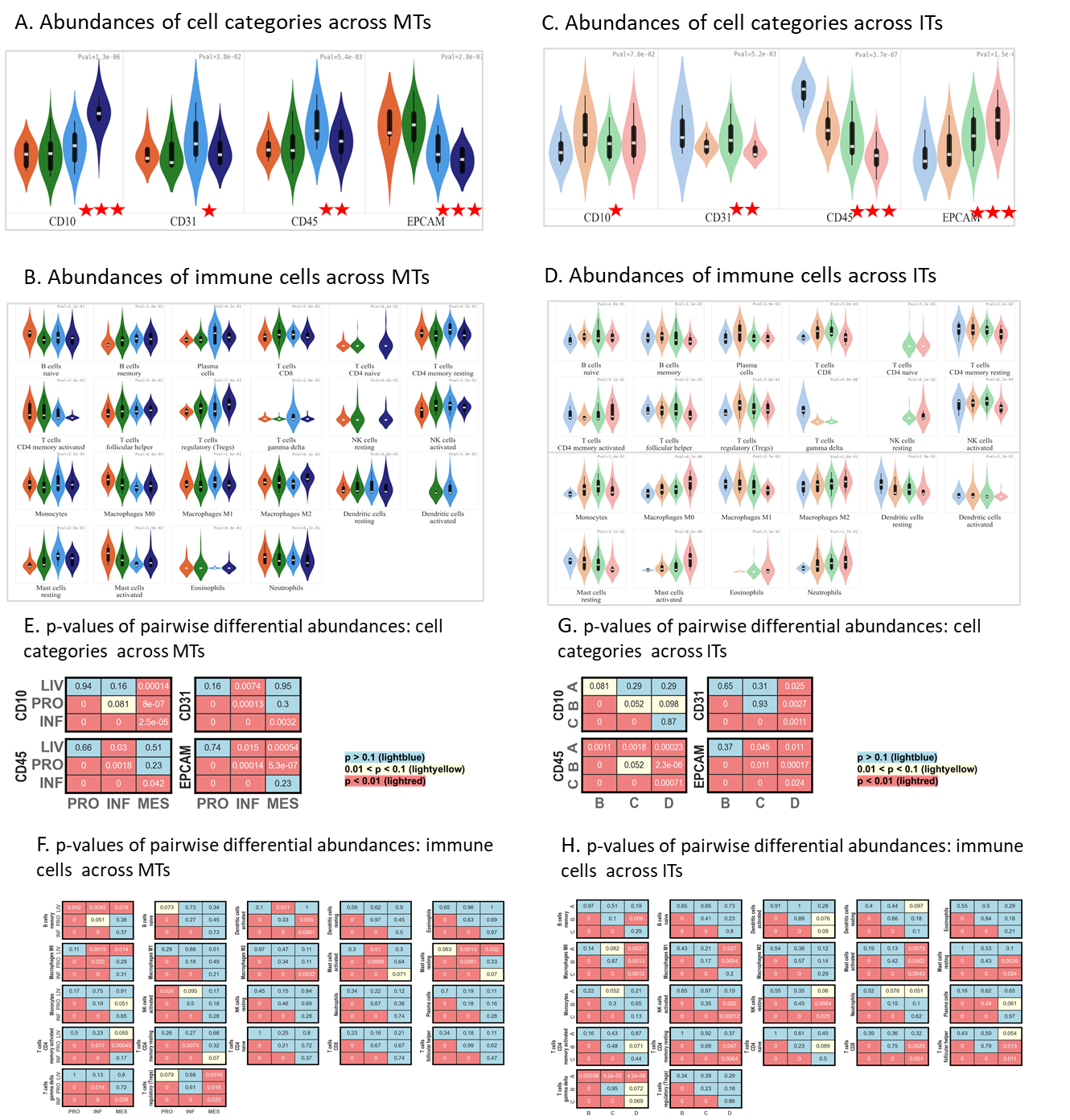


**Figure S16:** **Comparison of cell type fractions between MT (A and B) and between IT (C and D) for cell type categories** EPCAM (epithelial tumor cells), CD45 immune cells, CD10 (fibroblasts), CD31 (endothelial cells) (A, B), and LM22 immune cells (B, D). P-values are calculated using Kruskal-Wallis test statistics across the respective groups. (E-H), p-values of all pairwise comparisons (Mann-Whitney U-test) are shown in (E) for cell categories between MTs, (F) for immune cells (LM22) between MTs, (G) for cell categories between ITs, (H) for immune cells (LM22) between ITs. MTs pairwise comparisons: with the exception of LIV-vs-PRO, all differential abundance comparisons were statistically significant, particularly CD45 and CD10 had significantly different abundance between INF and MES. IT pairwise comparisons: CD45 and EPCAM had significantly different abundance for all comparisons with the exception of the EPCAM score between interferon-high cluster A vs cluster B.


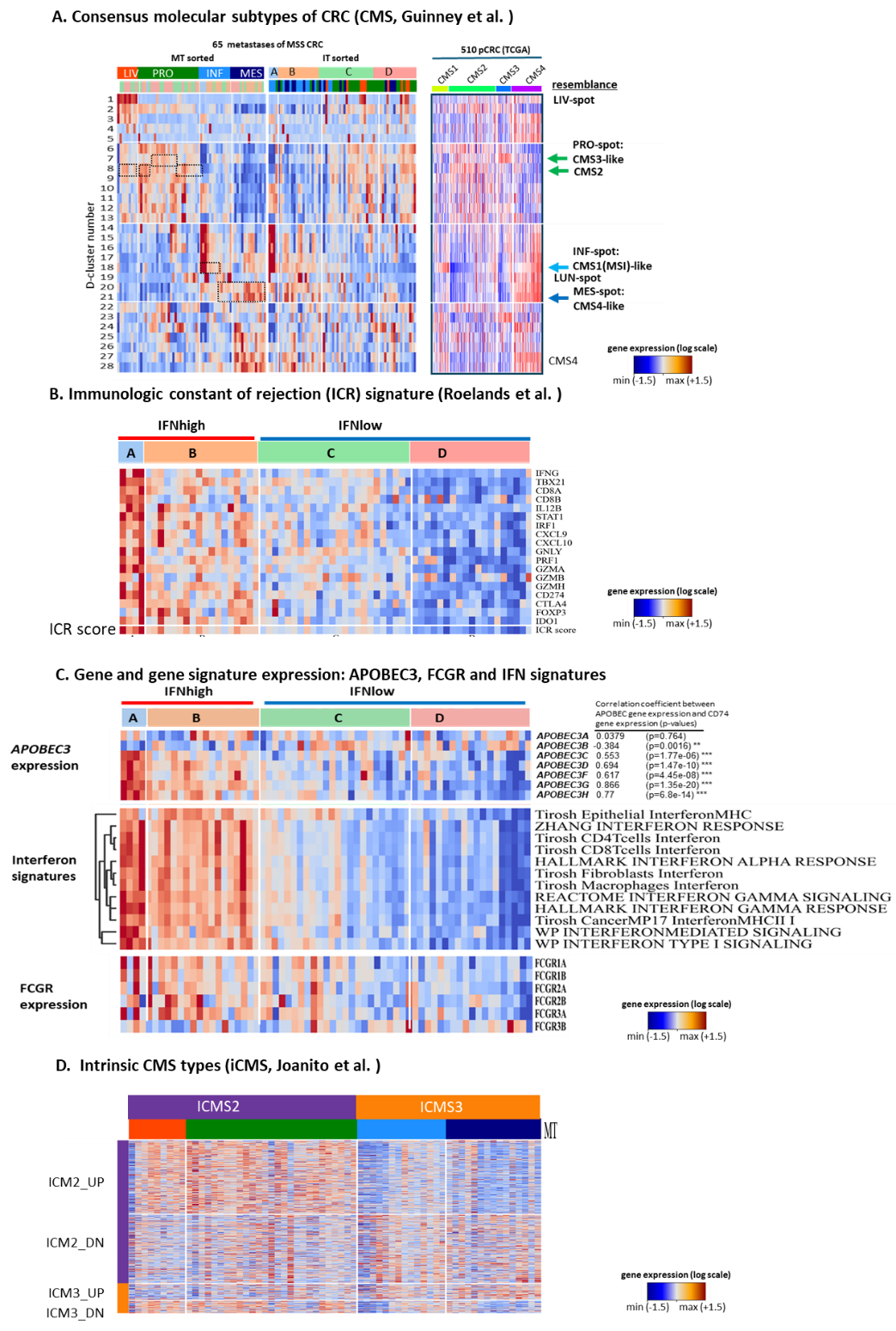


**Figure S17:** **Stratification of the tumors into alternative subtypes, clustered by interferon immunophenotypes (IT).** (A) Consensus molecular subtypes (CMS 1-4) were developed to classify CRC [43]. Left: heatmap of the expression scores of the 28 D-clusters segmenting the C-800-01 SOM by MT and IT. Right: heatmap of the expression scores of the same 28 D-clusters applied to the TCGA cohort of 510 CRC tumors. Samples are sorted by CMS1-CMS4 subtypes. (B) A cytotoxicity gene expression signature, called Immunologic Constant of Rejection (ICR) was shown to capture the presence of clonally expanded, tumor-enriched T cell clones and to outperform conventional prognostic molecular CRC biomarkers, such as the CMS and the MSI/MSS classifications [44, 45]. The ICR score showed high concordance with the interferon immunophenotypes [46]. (C) Expression of *APOBEC3*-type genes, of diverse interferon gene expression signatures, and of immunoglobulin gamma Fc region receptors (FCGR) genes followed the interferon scale used for immunotyping. Functional enrichment analysis showed that upregulation of *APOBEC3-B* associated with activation of proliferative functions while upregulation of *APOBEC3-C/D/F/G/H* was associated with activation of immune functions and, particularly, of T-cells. (D) CRC can be divided into two intrinsic consensus molecular subtypes, intrinsic CMS2 (iCMS2) and intrinsic CMS3 (iCMS3), based on single cell transcriptomes of epithelial tumor cells [47] which show resemblance with the transcriptomes of the LIV/PRO and INF/MES MTs in our cohort, respectively, using signatures taken from [47]. iCMS2 and iCMS3 are independent of microsatellite instability (MSI) status and one third of MSS CRC tumors were described to be transcriptionally more similar with MSI than to other MSS tumors [47]. Our MSS mCRC MES and INF tumors transcriptionally resembled the MSI-like iCMS3 subtype, while LIV and PRO showed similarities with the non-immunogenic iCMS2 signature.


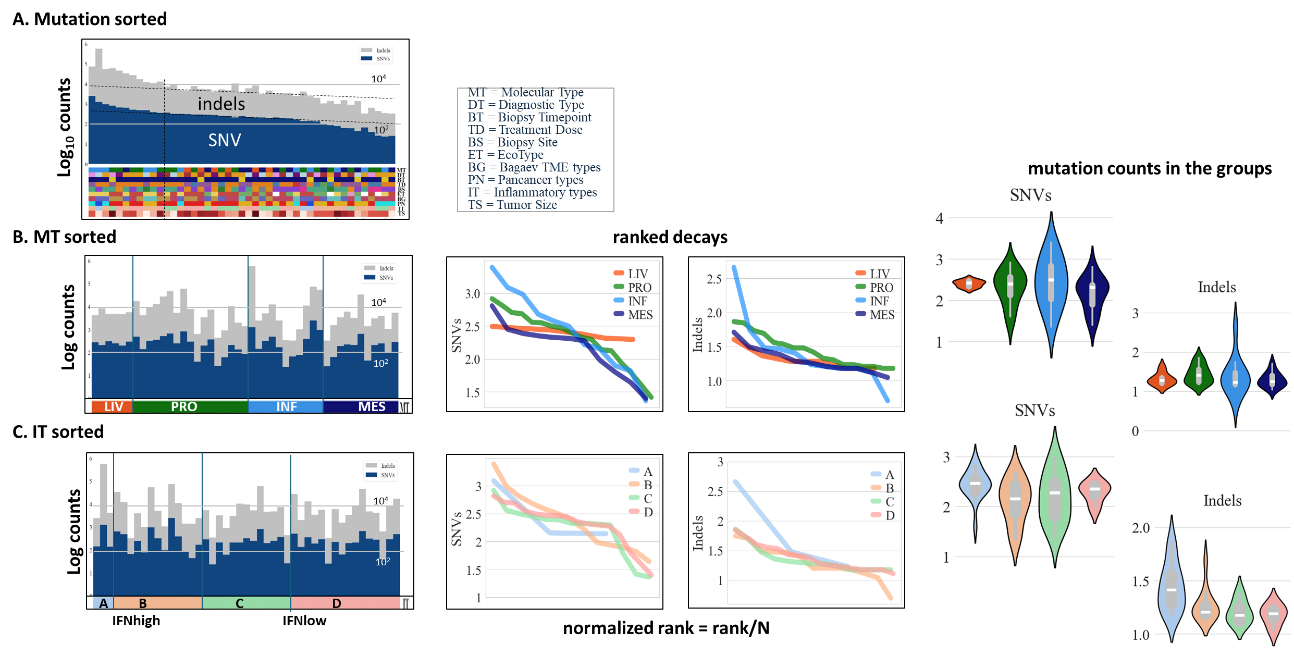


**Figure S18:** **Mutation counts of the C-800-01 tumors.** Number of small somatic variants: single nucleotide variants (SNV) and small indels (insertion deletions). (A) sorted by value: all samples had low numbers of small somatic variants consistent with their MSS status. (B) Sorted by MT. (C) Sorted by interferon IT. Overall, MTs and ITs were independent of small variant mutational burden, although there was a non-statistically significant trend for samples with high SNV/indels to be in the INF MT and the interferon-high cluster A IT.


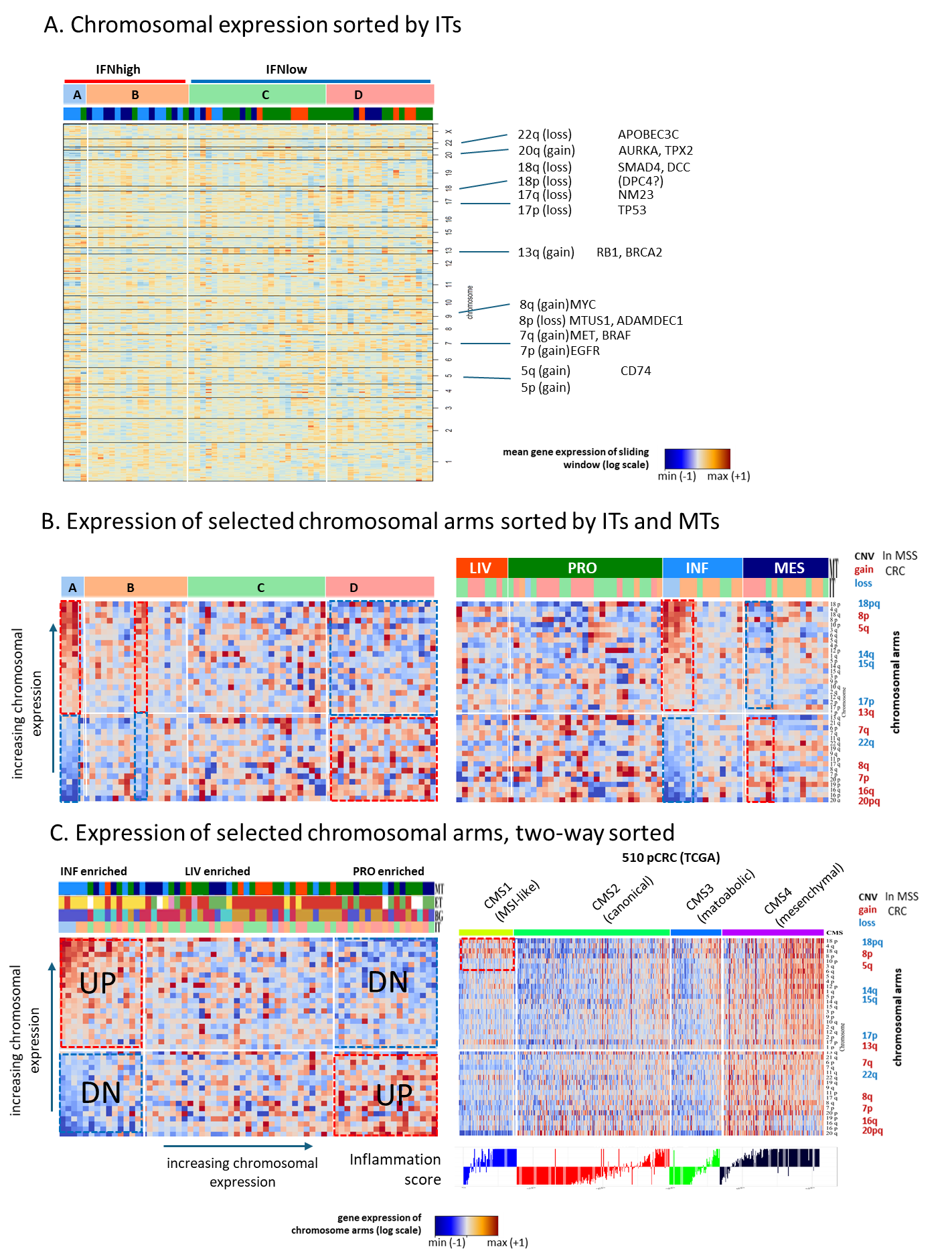


**Figure S19:** **Chromosomal expression (copy number variations [CNV]) association with tumor subtypes.** (A) Chromosomal expression (entire chromosome) was estimated by averaging expression over a sliding window of 50 genes along the entire chromosome. Brown indicates overexpression and blue indicates underexpression as a proxy for copy number gains and losses, respectively. Selected chromosomal regions prone for CNV in CRC are indicated. (B) Alternatively, the mean gene expression of selected chromosome-arms were calculated as mean gene expression averaged over all genes located at the respective chromosome arm, and sorted by interferon immunophenotype (left) and molecular types (right) (C left) Same heatmap sorted by sample cross-chromosome arm average expression and chromosome arm cross-sample average expression. (C right) Chromosome arm expression in the TCGA CRC cohort, sorted by CMS subtype and inflammation score. Overall, our analysis shows that CRC-typical CNV-patterns are maintained in the metastases and that specific chromosomal arm CNVs (as approximated by gene expression) were associated with a more immune inflamed (interferon-high, INF) transcriptomic subtype.


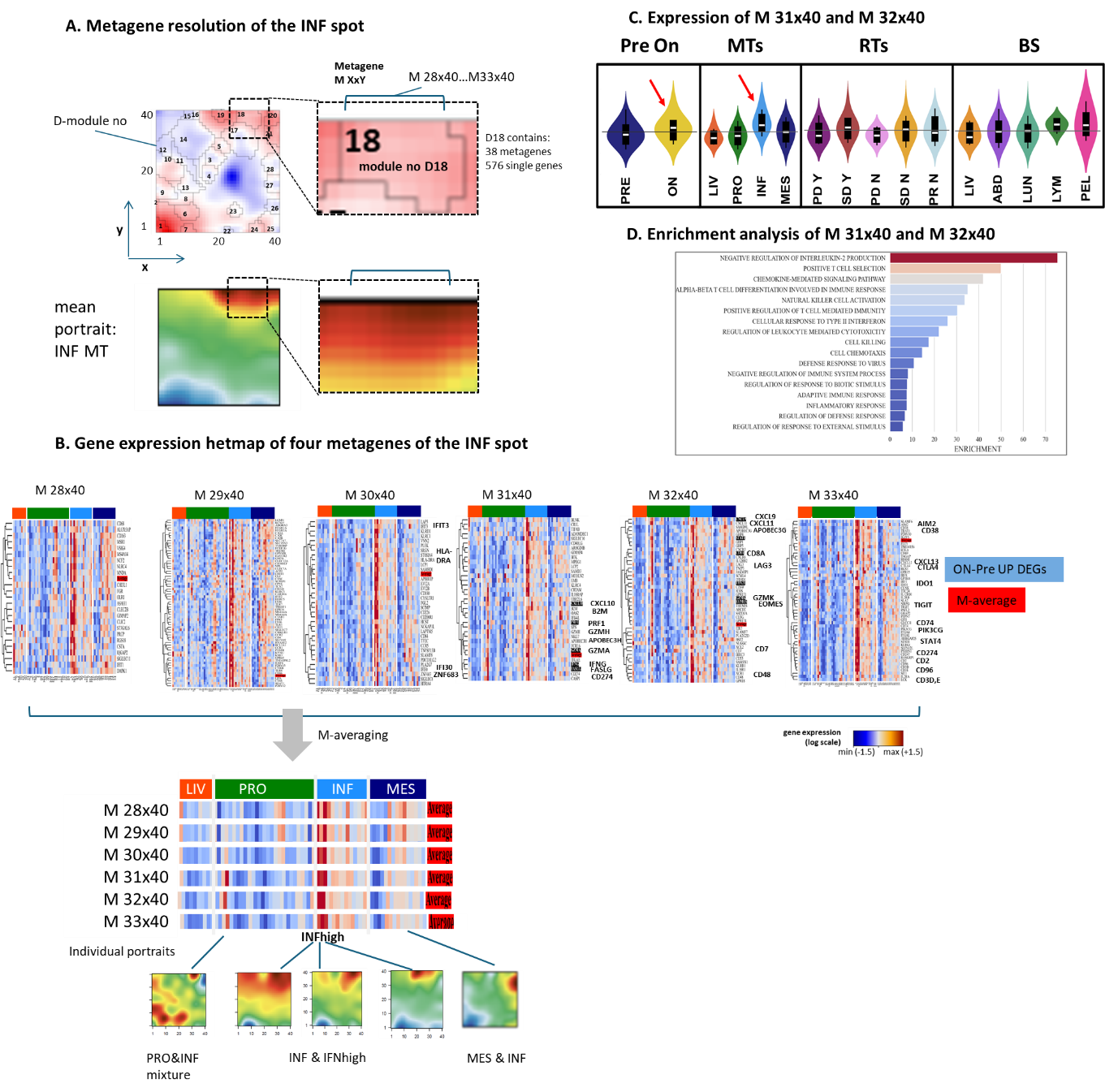


**Figure S20: Metagene resolution of the INF-spot** (A) The INF module is comprised by the D18 module consisting of 38 metagenes (pixels) and 576 single genes. Metagenes are identified by their horizontal x vertical-coordinates in the SOM image, e.g., M28x40 to M33x40 form the upper row of metagenes in D18. (B) Expression heatmaps of these metagenes, showing expression pattern across MTs. . (C) Metagenes M31x40 and M32x40 showed upregulation in on-treatment samples compared to paired pre-treatment samples (Pre-vs-ON) , and in the INF MT (red arrows). (D) Enrichment analysis of the genes of these two metagenes provide functional contexts related to immune response including interleukin production, chemokine and interferon signaling, and NK activation.


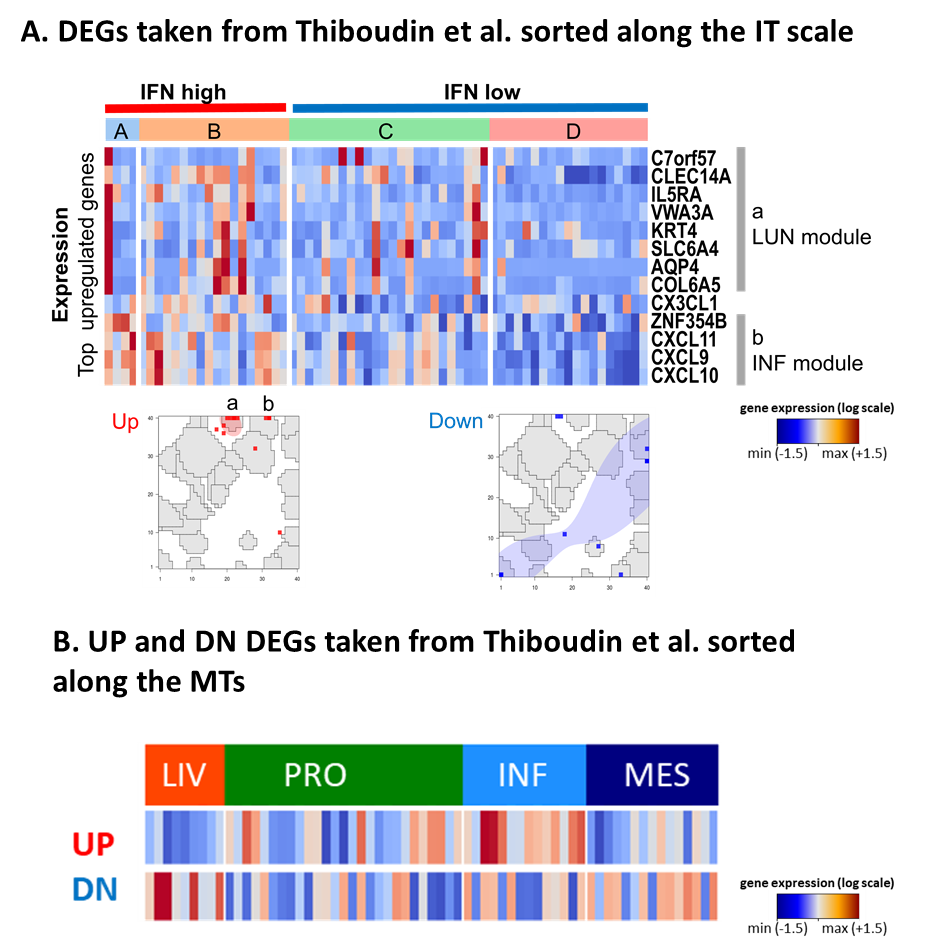


**Figure S21:** **Differentially expressed genes taken from Thiboudin et al. [48].** This study analyzed 36 unresectable MSS mCRC under combined anti–*PD-1* and anti–*CTLA-4* as well as neoadjuvant (FOLFIRI) therapy [48]. (A) Differentially expressed genes when mapped to the C-800-01 SOM consistently reflect an ICI-induced shift towards IE-states, however, with less pronounced correlation with the IT-scale, including accumulation in the INF- and LUN-modules, which suggests biased selection of LUN metastases. (B) Comparison of UP and DN regulated genes indicates accumulation of the former genes in the INF MT and of the latter ones in LIV and MES MTs.


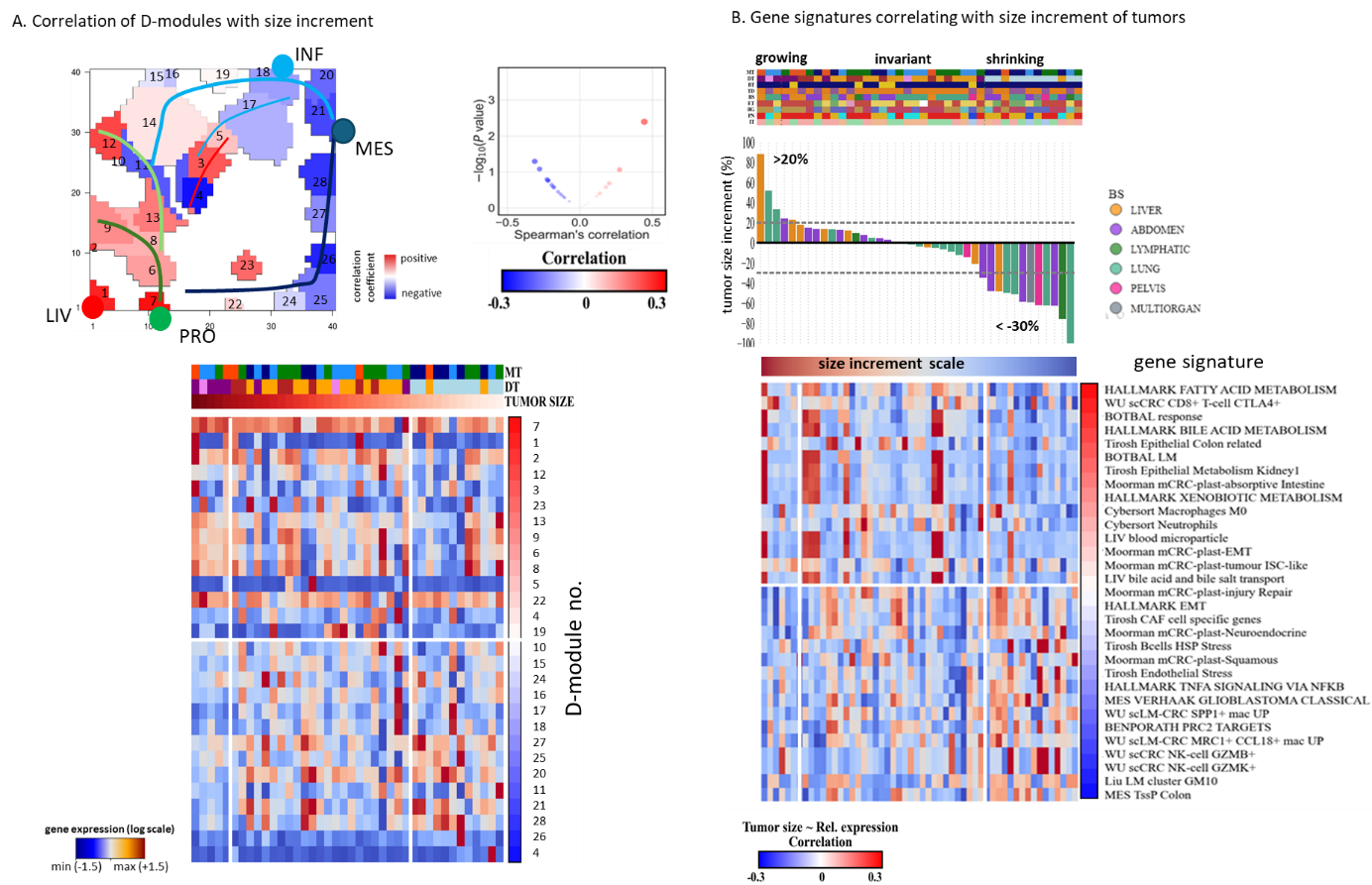


**Figure S22:** **Correlation of tumor size increment with transcriptomic signatures.** (A) Correlations with D-modules D1-28 indicate strongest positive (Pearson’s) with the PRO-spot D7, D2 (oxphos), and D12. Negative correlation (e.g., correlation with tumor size decay) was found for D-modules D11, D18, D20, D21, and D28. The significance scale is shown on the right. (B) The size-increment histogram and its correlation with expression scores of functional gene sets: Liver tissue and colon epithelium related functions positively correlate with tumor size, while immunogenic functions such as enrichment of NK cells and Hallmark TNFA-signaling via NFkB as well as EMT and PRC2 targets negatively correlate, meaning that they associate with shrinkage of the tumor.


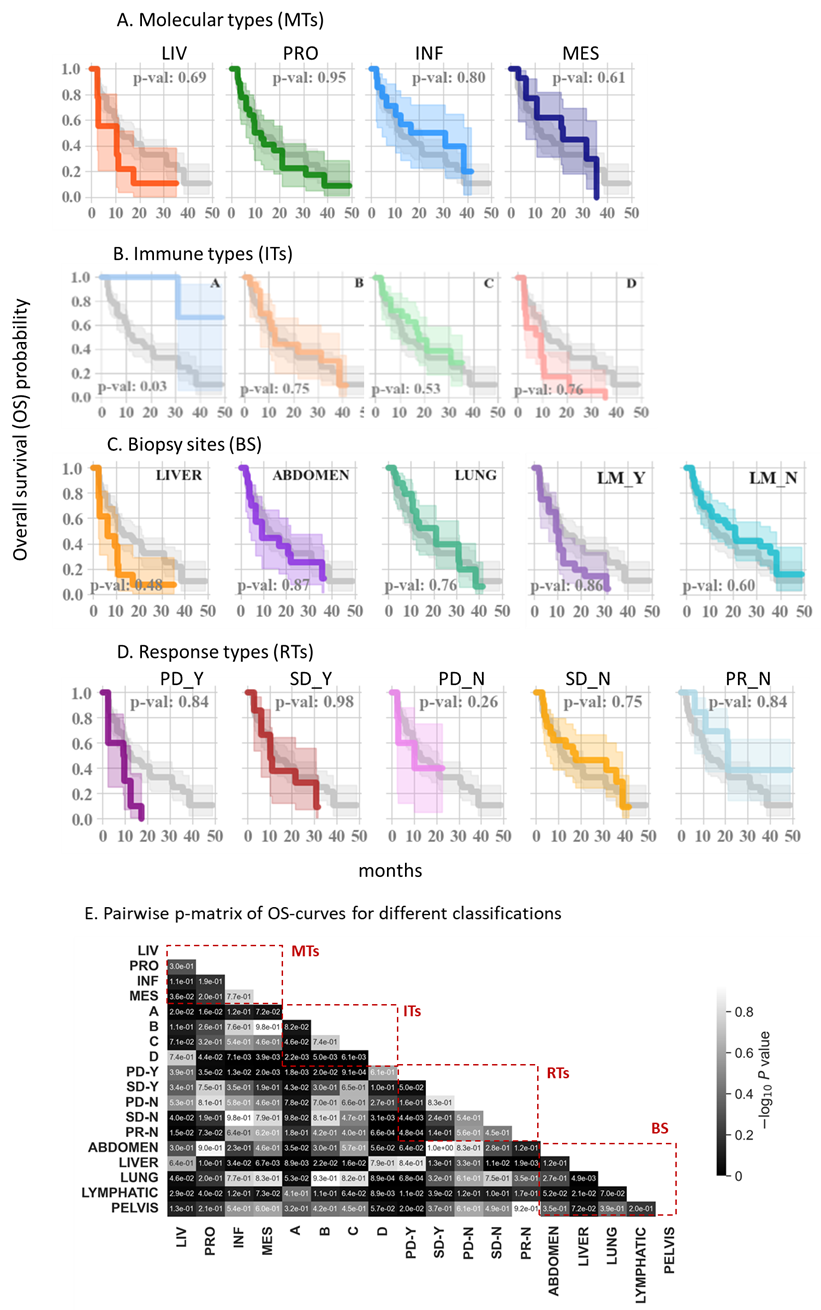


**Figure S23:** (A)-(D). **Overall survival Kaplan–Meier curves** (p-values on graphs correspond to the comparison of each group with the overall cohort). (E) p-values of all pairwise comparisons of OS-curves (log rank test).

.


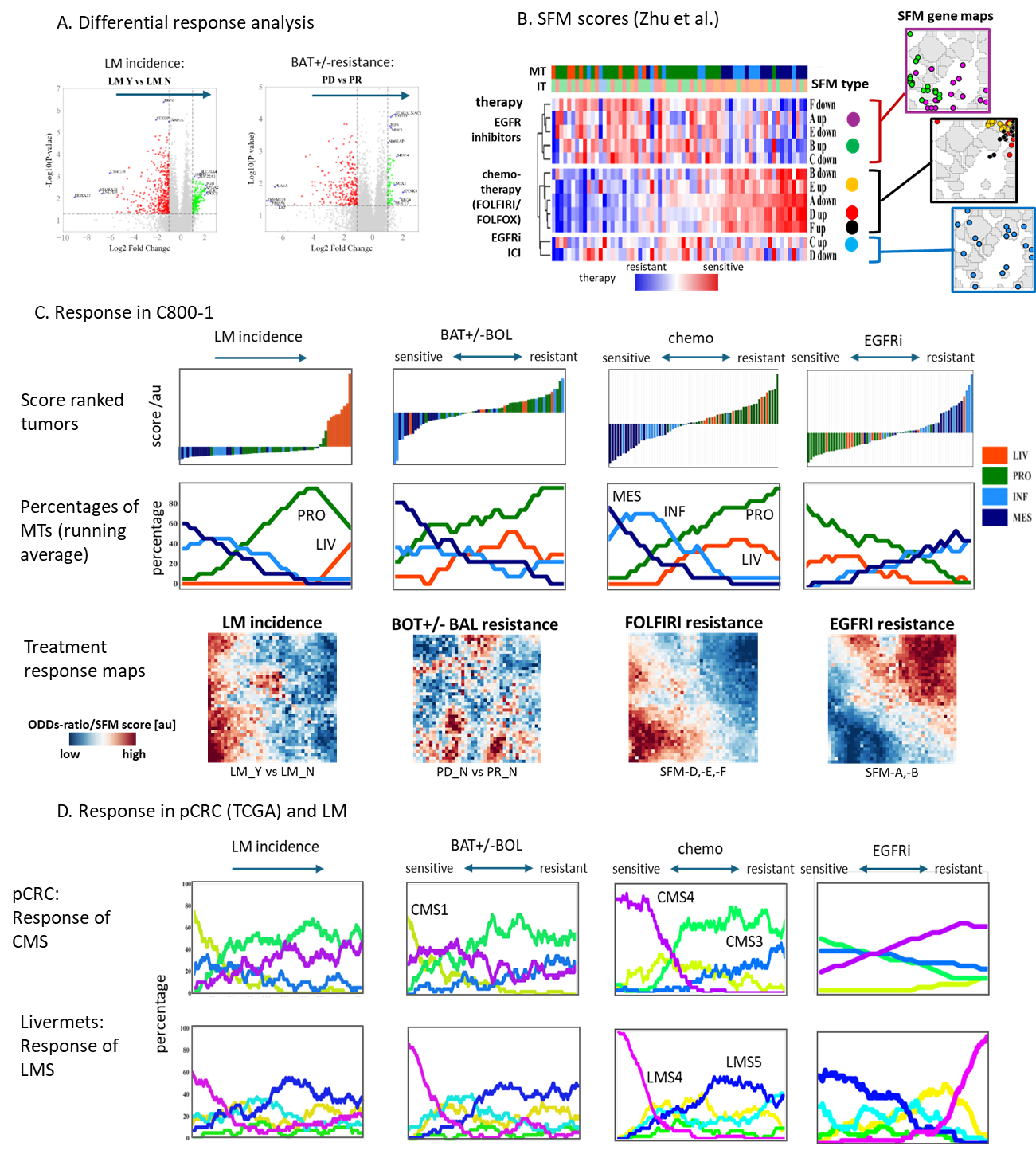


**Figure S24:** **Score analysis for the incidence of liver metastases (LM) and for resistance or sensitivity to BOT±BAL immunotherapy, chemotherapy (FOLFIRI/FOLFOX), and EGFR inhibitors.** (A) Differential response analysis was conducted by comparing transcriptomes of tumors with versus without active liver metastases (LM_Y vs. LM_N), and by comparing progressive disease versus partial responders among tumors without LM (PD_N vs. PR_N). Log-transform of the mean expression levels of the differentially upregulated genes were used. (B) Therapy-sensitivity gene-expression scores were adopted from Zhu et al. [49], who defined the "Signature associated with FOLFIRI resistance and Microenvironment" (SFM) in CRC patients. The SFM signature partitions into six clusters (SFM-A to SFM-F), each comprising an up- and down-regulated gene set. These clusters are associated with sensitivity to FOLFIRI and FOLFOX chemotherapy (SFM-D, -E, -F up) and to EGFR inhibitors (SFM-A, -B, -C up). On the C-800-01 SOM map, the SFM-A genes mostly co-localized with the PRO module and interferon-low IT, SFM-B genes with the cell-cycle/MYC-targets and metabolic modules, and SFM-C genes were located across broader regions associated with immune and fibrotic functions. Conversely, SFM-D and SFM-E genes co-localized with the MES and INF modules respectively, and SFM-F with both. (C) Tumors ranked by their respective scores are displayed with their MT classification (top row), running-average percentages (middle row), and odds-ratio treatment score maps (bottom rows). BOT±BAL and chemo/FOLFIRI scores indicate treatment sensitivity in MES and INF tumors and resistance in PRO tumors, whereas this pattern is reversed for EGFR-inhibitor treatment. The treatment maps highlight spatial detail of potential therapeutic response across the transcriptomic landscape. (D) We applied these scores to pCRC (TCGA) and LM transcriptomes [25] and ranked tumors according to increasing score values. Samples with low LM-incidence scores and high BOT±BAL sensitivity scores were enriched in inflammatory CMS1 subtypes . High chemo/FOLFIRI sensitivity scores were observed in CMS4 tumors and high chemo/FOLFIRI resistance scores in CMS3 tumors, whereas EGFR-inhibitor sensitivity displayed the opposite trend.

# References

1 Chand D, Savitsky DA, Krishnan S, Mednick G, Delepine C, Garcia-Broncano P *et al*. Botensilimab, an Fc-Enhanced Anti-CTLA-4 Antibody, Is Effective against Tumors Poorly Responsive to Conventional Immunotherapy. *Cancer Discov* 2024; 14: 2407–2429.

2 Bullock AJ, Schlechter BL, Fakih MG, Tsimberidou AM, Grossman JE, Gordon MS *et al*. Botensilimab plus balstilimab in relapsed/refractory microsatellite stable metastatic colorectal cancer: a phase 1 trial. *Nat Med* 2024; 30: 2558–2567.

3 Wilky BA, Schwartz GK, Gordon MS, El-Khoueiry AB, Bullock AJ, Henick B *et al*. Botensilimab (Fc-enhanced anti–cytotoxic lymphocyte-association protein-4 antibody) Plus Balstilimab (anti–PD-1 antibody) in Patients With Relapsed/Refractory Metastatic Sarcomas. *Journal of Clinical Oncology* 2025; 43: 1358–1368.

4 Löffler-Wirth H, Kalcher M, Binder H. oposSOM: R-package for high-dimensional portraying of genome-wide expression landscapes on bioconductor. *Bioinformatics* 2015; 31: 3225–3227.

5 Binder H, Wirth H. Analysis of large-scale OMIC data using Self Organizing Maps. In: Khosrow-Pour M (ed). *Encyclopedia of Information Science and Technology Third Edition*. IGI global, 2014, pp 1642–1654.

6 Wirth H, Löffler M, von Bergen M, Binder H. Expression cartography of human tissues using self organizing maps. *BMC Bioinformatics* (journal article) 2011; 12: 306.

7 Schmidt M, Hopp L, Arakelyan A, Kirsten H, Engel C, Wirkner K *et al*. The Human Blood Transcriptome in a Large Population Cohort and Its Relation to Aging and Health. *Frontiers in Big Data* (Original Research) 2020; 3.

8 Wirth H, von Bergen M, Binder H. Mining SOM expression portraits: Feature selection and integrating concepts of molecular function *BioData Mining* 2012; 5:18.

9 Hopp L, Wirth H, Fasold M, Binder H. Portraying the expression landscapes of cancer subtypes: A glioblastoma multiforme and prostate cancer case study. *Systems Biomedicine* 2013; 1: 99–121.

10 Loeffler-Wirth H, Kreuz M, Hopp L, Arakelyan A, Haake A, Cogliatti SB *et al*. A modular transcriptome map of mature B cell lymphomas. *Genome Medicine* (journal article) 2019; 11: 27.

11 Willscher E, Hopp L, Kreuz M, Schmidt M, Hakobyan S, Arakelyan A *et al*. High-Resolution Cartography of the Transcriptome and Methylome Landscapes of Diffuse Gliomas. *Cancers* 2021; 13: 3198.

12 Newman AM, Liu CL, Green MR, Gentles AJ, Feng W, Xu Y *et al*. Robust enumeration of cell subsets from tissue expression profiles. *Nature Methods* (Article) 2015; 12: 453.

13 Newman AM, Steen CB, Liu CL, Gentles AJ, Chaudhuri AA, Scherer F *et al*. Determining cell type abundance and expression from bulk tissues with digital cytometry. *Nat Biotechnol* 2019; 37: 773–782.

14 White BS, de Reyniès A, Newman AM, Waterfall JJ, Lamb A, Petitprez F *et al*. Community assessment of methods to deconvolve cellular composition from bulk gene expression. *Nature Communications* 2024; 15: 7362.

15 Nguyen H, Nguyen H, Tran D, Draghici S, Nguyen T. Fourteen years of cellular deconvolution: methodology, applications, technical evaluation and outstanding challenges. *Nucleic Acids Research* 2024; 52: 4761–4783.

16 Li H, Zhou J, Li Z, Chen S, Liao X, Zhang B *et al*. A comprehensive benchmarking with practical guidelines for cellular deconvolution of spatial transcriptomics. *Nature Communications* 2023; 14: 1548.

17 Luca BA, Steen CB, Matusiak M, Azizi A, Varma S, Zhu C *et al*. Atlas of clinically distinct cell states and ecosystems across human solid tumors. *Cell* 2021; 184: 5482–5496.e5428.

18 Yoshihara K, Shahmoradgoli M, Martínez E, Vegesna R, Kim H, Torres-Garcia W *et al*. Inferring tumour purity and stromal and immune cell admixture from expression data. *Nature Communications* 2013; 4: 2612.

19 Loeffler-Wirth H, Reikowski J, Hakobyan S, Wagner J, Binder H. oposSOM-Browser: an interactive tool to explore omics data landscapes in health science. *BMC Bioinformatics* 2020; 21: 465.

20 Rousseeuw PJ. Silhouettes: A graphical aid to the interpretation and validation of cluster analysis. *Journal of Computational and Applied Mathematics* 1987; 20: 53–65.

21 Gerber T, Willscher E, Loeffler-Wirth H, Hopp L, Schadendorf D, Schartl M *et al*. Mapping heterogeneity in patient-derived melanoma cultures by single-cell RNA-seq. *Oncotarget* 2017; 8: 846–862.

22 Avagyan S, Binder H. Subtyping or not subtyping— Quo vadis for precision medicine of colorectal cancer. *Translational cancer research* 2023.

23 Loeffler-Wirth H, Kreuz M, Schmidt M, Ott G, Siebert R, Binder H. Classifying Germinal Center Derived Lymphomas-Navigate a Complex Transcriptional Landscape. *Cancers* 2022; 14: 3434.

24 Moosavi SH, Eide PW, Eilertsen IA, Brunsell TH, Berg KCG, Røsok BI *et al*. De novo transcriptomic subtyping of colorectal cancer liver metastases in the context of tumor heterogeneity. *Genome Medicine* 2021; 13: 143.

25 Ashekyan O, Shahbazyan N, Bareghamyan Y, Kudryavzeva A, Mandel D, Schmidt M *et al*. Transcriptomic Maps of Colorectal Liver Metastasis: Machine Learning of Gene Activation Patterns and Epigenetic Trajectories in Support of Precision Medicine. *Cancers* 2023; 15: 3835.

26 Liu Z, Weng S, Dang Q, Xu H, Ren Y, Guo C *et al*. Gene interaction perturbation network deciphers a high-resolution taxonomy in colorectal cancer. *eLife* 2022; 11: e81114.

27 Yang S, Qian L, Li Z, Li Y, Bai J, Zheng B *et al*. Integrated Multi-Omics Landscape of Liver Metastases. *Gastroenterology* 2023; 164: 407–423.e417.

28 Liu J, Cho YB, Hong HK, Wu S, Ebert PJ, Bray SM *et al*. Molecular dissection of CRC primary tumors and their matched liver metastases reveals critical role of immune microenvironment, EMT and angiogenesis in cancer metastasis. *Scientific Reports* 2020; 10: 10725.

29 Liberzon A, Birger C, Thorvaldsdóttir H, Ghandi M, Mesirov Jill P, Tamayo P. The Molecular Signatures Database Hallmark Gene Set Collection. *Cell Systems* 2015; 1: 417–425.

30 Ben-Porath I, Thomson MW, Carey VJ, Ge R, Bell GW, Regev A *et al*. An embryonic stem cell-like gene expression signature in poorly differentiated aggressive human tumors. *Nat Genet* (10.1038/ng.127) 2008; 40: 499–507.

31 Gavish A, Tyler M, Greenwald AC, Hoefflin R, Simkin D, Tschernichovsky R *et al*. Hallmarks of transcriptional intratumour heterogeneity across a thousand tumours. *Nature* 2023; 618: 598–606.

32 Wu Y, Yang S, Ma J, Chen Z, Song G, Rao D *et al*. Spatiotemporal Immune Landscape of Colorectal Cancer Liver Metastasis at Single-Cell Level. *Cancer Discov* 2022; 12: 134–153.

33 Ernst J, Kellis M. Chromatin-state discovery and genome annotation with ChromHMM. *Nature Protocols* 2017; 12: 2478–2492.

34 Hebenstreit D, Fang M, Gu M, Charoensawan V, van Oudenaarden A, Teichmann SA. RNA sequencing reveals two major classes of gene expression levels in metazoan cells. *Mol Syst Biol* (10.1038/msb.2011.28) 2011; 7.

35 Shang J, Jiang H, Zhao Y, Yang J, Lin Y, Zhang N *et al*. Molecular subtyping of stage I lung adenocarcinoma via molecular alterations in pre-invasive lesion progression. *Journal of Translational Medicine* 2025; 23: 263.

36 Dai W, Guo C, Wang Y, Li Y, Xie R, Wu J *et al*. Identification of hub genes and pathways in lung metastatic colorectal cancer. *BMC Cancer* 2023; 23: 323.

37 Lenos KJ, Bach S, Ferreira Moreno L, ten Hoorn S, Sluiter NR, Bootsma S *et al*. Molecular characterization of colorectal cancer related peritoneal metastatic disease. *Nature Communications* 2022; 13: 4443.

38 Yun K, Merrie AEH, Gunn J, Phillips LV, McCall JL. Keratin 20 is a specific marker of submicroscopic lymph node metastases in colorectal cancer: validation by K-RAS mutations. *The Journal of Pathology* 2000; 191: 21–26.

39 Feng X, Wang Q. Keratin-15 high expression links with lymph node metastasis and poor survival prognosis in epithelial ovarian cancer patients. *Discover oncology* 2024; 15: 555.

40 Ha YJ, Park S-H, Tak KH, Lee JL, Kim CW, Kim J-H *et al*. CILP2 is a potential biomarker for the prediction and therapeutic target of peritoneal metastases in colorectal cancer. *Scientific Reports* 2024; 14: 12487.

41 Lenos KJ, Bach S, Ferreira Moreno L, Ten Hoorn S, Sluiter NR, Bootsma S *et al*. Molecular characterization of colorectal cancer related peritoneal metastatic disease. *Nat Commun* 2022; 13: 4443.

42 Chaintreuil P, Kerreneur E, Bourgoin M, Savy C, Favreau C, Robert G *et al*. The generation, activation, and polarization of monocyte-derived macrophages in human malignancies. *Front Immunol* 2023; 14: 1178337.

43 Guinney J, Dienstmann R, Wang X, de Reynies A, Schlicker A, Soneson C *et al*. The consensus molecular subtypes of colorectal cancer. *Nat Med* (Analysis) 2015; 21: 1350–1356.

44 Roelands J, Ploeg Mvd, Ijsselsteijn ME, Dang H, Boonstra JJ, Hardwick JCH *et al*. Transcriptomic and immunophenotypic profiling reveals molecular and immunological hallmarks of colorectal cancer tumourigenesis. *Gut* 2023; 72: 1326–1339.

45 Roelands J, Kuppen PJK, Ahmed EI, Mall R, Masoodi T, Singh P *et al*. An integrated tumor, immune and microbiome atlas of colon cancer. *Nat Med* 2023; 29: 1273–1286.

46 Acha-Sagredo A, Andrei P, Clayton K, Taggart E, Antoniotti C, Woodman CA *et al*. A constitutive interferon-high immunophenotype defines response to immunotherapy in colorectal cancer. *Cancer Cell* 2025; 43: 292–307.e297.

47 Joanito I, Wirapati P, Zhao N, Nawaz Z, Yeo G, Lee F *et al*. Single-cell and bulk transcriptome sequencing identifies two epithelial tumor cell states and refines the consensus molecular classification of colorectal cancer. *Nat Genetics* 2022; 54: 963–975.

48 Thibaudin M, Fumet JD, Chibaudel B, Bennouna J, Borg C, Martin-Babau J *et al*. First-line durvalumab and tremelimumab with chemotherapy in RAS-mutated metastatic colorectal cancer: a phase 1b/2 trial. *Nat Med* 2023; 29: 2087–2098.

49 Zhu X, Tian X, Ji L, Zhang X, Cao Y, Shen C *et al*. A tumor microenvironment-specific gene expression signature predicts chemotherapy resistance in colorectal cancer patients. *npj Precision Oncology* 2021; 5: 7.
